# Spatial attribution and policy design of rural entrepreneurship: Evidence from Anhui Province in China

**DOI:** 10.1371/journal.pone.0331419

**Published:** 2025-09-17

**Authors:** Xiaojuan Yang, Weiwei Li, Zhenyu Gao

**Affiliations:** 1 Civil & Architecture Engineering, Xi’an Technological University, Xi’an, China; 2 Urban-Rural Construction College, Guangxi Vocational University of Agriculture, Nanning, China; 3 The School of Art and Media Design, Nanchang Institute of Science and Technology, Nanchang, China; West Pomeranian University of Technology, POLAND

## Abstract

Rural entrepreneurship is a key way to combat rural decline and promote the revitalization of rural areas and their sustainability, and also a key area of research in agricultural and rural geography, economics, and management. We combined spatial econometrics models such as spatial clustering, cold and hot spot analysis, geographical weighted regression and Geodetector to carry out empirical research on geographical distribution of rural entrepreneurship in Anhui province, in an attempt to provide scientific basis for rural policy design, spatial planning and evidence-based decision-making. The findings showed an increasing trend of the spatial heterogeneity and autocorrelation of rural entrepreneurship in Anhui, with geographic clustering of high, medium and low values as well as cold and hot spots. And the diversification of rural entrepreneurship changes led to a very complex driving mechanism for the generation and evolution of rural entrepreneurship spatial patterns, and the factors showed significant spatial and composite effects. The enlightening value of the analysis results lies in the fact that rural entrepreneurship management not only needs to delineate geographical zones and design differentiated policies, but also needs to jointly build rural entrepreneurship alliances in similar or adjacent areas to integrate regional entrepreneurial resources. In addition, rural entrepreneurship management should be guided according to the situation, and policy design should take into account both quantity and speed control, with establishment of policy combinations based on the spatial and composite effects of different factors.

## Introduction

### Research background

Entrepreneurship is the practice of entrepreneurs and their partners through the integration of resources they have, and a process of establishing a new business or starting a new business to create greater economic or social value. Wortman was the first scholar to propose the concept of rural entrepreneurship, which he described as the process of creating new organizations in the larger rural environment to provide new products or services, develop new markets, or utilize new technologies [1,2]. Rural entrepreneurship has become a key driver of rural revitalization, especially rural economic sustainability, and the study of rural entrepreneurial ecosystems is of great value [3].

With the development of rural entrepreneurship in China in full swing and the issuance of supportive policies, rural entrepreneurship research has become an emerging hot spot [4]. The General Office of the State Council of China has issued the Opinions on Supporting Migrant Workers and Other Groups in Returning to Their Hometowns for Entrepreneurship and the Opinions on Supporting Returnees and Rural Migrants in Entrepreneurship and Innovation to Promote the Integrated Development of Primary, Secondary, and Tertiary Industries in Rural Areas, providing policy support for migrant workers, university graduates, demobilized soldiers, scientific and technical personnel, and other returnees and rural migrants to engage in entrepreneurship and innovation in rural areas. The Ministry of Agriculture and Rural Affairs, the Ministry of Science and Technology, the Ministry of Finance and other government departments have jointly issued the Opinions on Promoting the Construction of Return-to-Rural Entrepreneurship Parks to Enhance Rural Entrepreneurship and Innovation Levels and the Opinions on Further Promoting Return-to-Rural Entrepreneurship Work. These documents outline a policy list for rural entrepreneurship support, covering tax and fee reductions, land and insurance support, investment and financing and loan guarantees, talent training and development, the construction of entrepreneurial mentor teams, and the establishment of entrepreneurial service platforms and park carriers. According to the statistics of the National Development and Reform Commission of China, a total more than 11.2 million individuals returned to their hometowns for businesses as of 2022, with migrant workers accounting for 70%.

Of note, studies available have shown that entrepreneurship is highly heterogeneous, and the entrepreneurial process and results are closely related to the environment. Therefore, to meet the needs of rural entrepreneurship management policy design, it is of great theoretical significance and practical value to conduct in-depth research on the changing trend, geographical distribution law and influencing factors of rural entrepreneurial activities based on the heterogeneity of the geographical environment of rural entrepreneurship, coupled with the complexity of the evolution process of rural entrepreneurship, by means of multidisciplinary theories and methods [5].

### Literature review

The research on rural entrepreneurship is mainly conducted by theories and methods of economics and management, with the research paradigms of geography on the rise. The core issue that economists are generally concerned about when analyzing rural entrepreneurship using the paradigm of rural and agricultural economic theory is what role entrepreneurs play in resource allocation in rural areas, and what impact entrepreneurial activities have on the sustainability of rural economy and what the mechanism of action is. From the perspective of rural entrepreneurship to optimize and reconfigure rural resource allocation, economists regard rural entrepreneurs as the bearers of uncertainty risks in rural revitalization and high-quality development (Knight), the organizers and coordinators of development resources such as rural land – labor – capital – technology – data (Coase), and the arbitrageurs in an unbalanced market (Kirzner). From the perspective that rural entrepreneurship influences or promotes high-quality rural economic development, economists view them as creative market disruptors capable of breaking market equilibrium and promoting revolutionary technological innovation and production progress. Rural entrepreneurship plays a crucial role in promoting rural revitalization, especially economic sustainability [6,7]. Candelario-Moreno explained the dynamics of rural entrepreneurship as a driver of endogenous development in the Spanish countryside, and clarified the influence of different factors on it [8]. Batool found a strong positive correlation between the dynamics of economic development in Pakistan’s Special Economic Zones, regions along the economic corridors and local rural entrepreneurship. Li found through empirical analysis using the multi period double difference method that the construction of innovation pilot projects significantly promoted agricultural and rural entrepreneurship, and its role was achieved by high-tech investment, credit support, technological innovation, and other means [9].

Under the management and geography paradigm, rural entrepreneurs have become rural populations who identify, evaluate, and utilize limited market and spatial opportunities in a given village. Management scholars focus their efforts on policy design and the key technologies of developing strategies or strategies and their realization paths, and are committed to providing a basis for rural entrepreneurial management. Meera proposed an integrated intervention strategy for community-based rural entrepreneurship, including governance based, ecology based, and business-based approaches [10]. Deller found in his empirical analysis of the United States that older immigrants have a stronger influence on rural entrepreneurship than younger immigrants [11]. Geography study focuses on the spatial generation process of rural entrepreneurship, and is committed to analyzing the distribution characteristics and evolution patterns of rural entrepreneurship in the geographic space. Focusing on analyzing the geographical distribution of entrepreneurial activities, geographers are committed to revealing the complex interactions between geography and entrepreneurship to provide a basis for entrepreneurial spatial arrangement or planning [12,13]. Wolff argued that geographic location and spatial context are important for entrepreneurial activities and proposes the concept of entrepreneurial activity continuum from a geographic distribution perspective by analyzing the comparative variability in geographic location of family firms and start-ups [14]. Yeung analyzed cross-border entrepreneurship based on economic geography and multinational corporation theory, and proposed the concept of entrepreneurial spatial relations [15].

The research gap is mainly the disconnection between the theoretical analysis of academic research on rural entrepreneurship and the knowledge needs of management practice, with scholars’ research focusing on individual case studies and government management based on overall policy design. Most of the current studies focus on the case analysis of rural entrepreneurial individuals and have established a relatively sophisticated knowledge map. Notably, rural entrepreneurship management and policies are designed for administrative regions rather than individual entrepreneurs, and there are a variety of rural entrepreneurs in any township, county (district), province (state), country or region. According to the theory of systems science, the whole is not equal to the simple sum of its parts. Therefore, rural entrepreneurship as a whole is not the simple sum of rural entrepreneurial individuals, and knowledge gained through individual studies hardly provides an accurate guide for overall decisions of high diversity and complexity. And although entrepreneurship management and geography studies have yielded fruitful results, most of them focus on urban entrepreneurship, manufacturing entrepreneurship, or high-tech entrepreneurship, with few on rural and agricultural entrepreneurship [16]. In this context, the scientific questions of this study are the following three:

First, how to scientifically evaluate the development level and evolution model of rural entrepreneurship in different regions? In this study, we constructed an evaluation index system for rural entrepreneurship and introduced traditional statistical and geographic statistical models to quantitatively analyze the changing trends and spatial distribution characteristics of rural entrepreneurship.

Second, what is the driving mechanism of the difference in the distribution and change of rural entrepreneurship in different regions? In this study, we quantitatively measured the influence mechanisms of different factors on the development and changes of rural entrepreneurship using PSR and spatial econometric models, including the nature, intensity, spatial effects, and interaction effects of multiple factors.

Third, what enlightenment will the research results provide for the policy design and spatial planning of rural entrepreneurship management? We conducted a superposition analysis of the current characteristics and influencing factors of rural entrepreneurship by matrix analysis, and proposed countermeasures and suggestions for rural entrepreneurship policy design based on spatial zoning and management strategies.

## Research design

### Study area

Among the more than 30 provincial-level administrative regions in China, rural entrepreneurship in Anhui Province is notably representative. The reasons for selecting Anhui Province as the study area include the following: On one hand, from the perspective of the composition and supply of entrepreneurs, migrant workers who return to their hometowns after working elsewhere are the main force of rural entrepreneurs in China. With the transfer of industries from Jiangsu and Zhejiang to Anhui, a large number of migrant workers from Anhui Province returned home to start businesses as early as 2013. On the other hand, from the perspective of the industrial environment and entrepreneurial demand, Anhui is the birthplace of China’s rural reform and a major agricultural province in China. It has a large rural population and a substantial agricultural industry scale, making the task of rural revitalization and development formidable, and the demand for rural entrepreneurship immense. Additionally, Anhui Province has formulated and implemented a large number of rural entrepreneurship policies, promoting rural entrepreneurship as a typical representative in the country. By the end of 2022, the number of migrant workers returning home to start businesses in Anhui Province reached 561300, and 285700 economic entities were established. According to the current administrative management system and main functional zone planning in China, the districts of prefecture level cities mainly undertake the functions of industrialization and urbanization, while counties and county-level cities are mainly responsible for agricultural modernization and rural revitalization. Anhui Province has 16 prefecture-level cities, including Hefei, Huaibei, Bozhou, Suzhou, Bengbu, Fuyang, Huainan, Chuzhou, Lu’an, Ma’anshan, Wuhu, Xuancheng, Tongling, Chizhou, Anqing, and Huangshan. These are urban areas with limited rural territories and a small population engaged in rural entrepreneurship, hence they are excluded from the study area. Therefore, the study area of this paper covers 9 county-level cities and 50 counties in Anhui, a total of 59 county-level administrative regions.

### Theoretical assumptions and technical roadmap

#### First hypothesis: rural entrepreneurship has significant spatial effects.

The generation and changes of entrepreneurial activities exhibit heterogeneity and complexity, showing significant differences for different groups, which has become a consensus in academia. On that basis, this study further hypothesizes that rural entrepreneurship also exhibits significant spatial heterogeneity in its geographical distribution, that is, entrepreneurial activities, processes and performance in different regions are significantly different. Besides, given that rural entrepreneurial activities are stimulated by accessible and especially successful entrepreneurial examples in neighboring areas, coupled with the fact that entrepreneurial activities involve the coordination of land, technology, labor, data and other resources., and that the resources between different regions are spatially related [17,18], this study further hypothesizes that rural entrepreneurship features spatial autocorrelation. Overall, this study proposes the hypothesis that rural entrepreneurship has spatial effects and exhibits significant spatial heterogeneity and autocorrelation. The enlightening value of this hypothesis is how to embed the spatial effect into the management of rural entrepreneurship to provide a basis for the design of differentiated management policies for rural entrepreneurship, the establishment of rural entrepreneurship alliances, and the spatial planning of rural entrepreneurship [19].

#### Second hypothesis: driving mechanism of rural revitalization has significant composite effects.

The rural context is highly heterogeneous and rural entrepreneurship is always under its influence, to a certain extent rural entrepreneurial activity can be regarded as the interactive process and result of the rural environment and entrepreneurs [20,21]. In addition, rural entrepreneurship is not an activity that entrepreneurs can accomplish independently, but requires the close cooperation of the government, banks, employees, village committees, other villagers, and upstream and downstream industry chain suppliers and sellers. Therefore, rural entrepreneurship management strategies must be proposed on the basis of rural scenarios and their influencing mechanisms to clarify the responses of different actors to rural entrepreneurial activities. The rural context is composed of multidimensional factors such as economy, society, culture, politics and policies, and ecology. This study hypothesizes that rural entrepreneurship is the result of collaborative actions of multiple subjects under the joint action of multiple factors. The enlightening value of this hypothesis is to design policies that are in essence compatible with the rural situation and the response of actors for the management strategy of rural entrepreneurship. Therefore, it is necessary to select the influencing factors from the perspective of rural entrepreneurship state and subject’s response, and to categorize the nature and intensity of each factor’s influence on rural entrepreneurship, as well as its spatial effect and interaction effect. The purpose of spatial effect analysis is to reveal the influence of geographical distribution on the nature and strength of each factor, while interaction effect analysis aims to show the mutual constraints or facilitating effects of different factors when they work together.

#### Theoretical framework and technical roadmap of rural revitalization.

**Fig 1:**
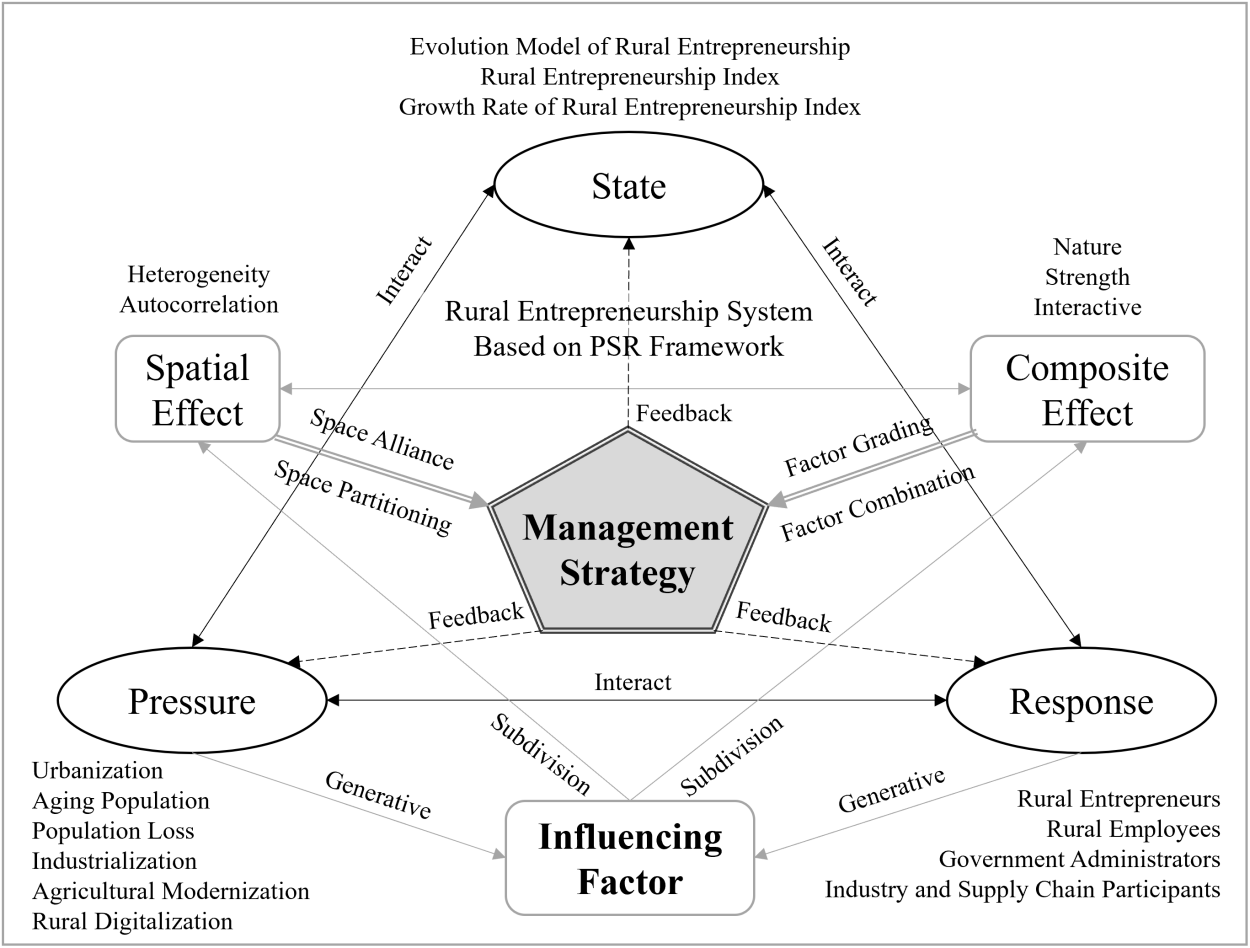
Theoretical Framework of rural entrepreneurship.

This paper integrates the two theoretical assumptions of spatial and composite effects of rural entrepreneurship and the evolution model, influencing factor and management strategy of rural entrepreneurship based on the PSR (Pressure-State-Response) model, to construct a new theoretical framework for rural entrepreneurship research. The PSR model, proposed by D. Rapport et al. and then improved and promoted by the International Organization for Economic Development and Cooperation, has become one of the most commonly used dynamic ones in sustainability research [22]. The use of PSR model to construct the rural entrepreneurship system can well help to show the interaction between the multi-factor pressure of rural entrepreneurship situation, the state of the generation and evolution of rural entrepreneurial activities, and the behaviors of multi-interested subjects in rural entrepreneurship. The fundamental need for most rural entrepreneurial activities is not for development but for survival. To this end, the state of rural entrepreneurship activities can be seen as the differentiated response of the rural entrepreneurial community to pressure in the context of rural revitalization, and the mutual feedback in the process to ultimately form a rural entrepreneurial system of dynamic evolution and interactive conduction. The study of the rural entrepreneurship state subsystem focuses on the evolution model, including both the rural entrepreneurship index and its growth rate. The rural entrepreneurial pressure subsystem focuses on the challenges faced or strategic goals that must be achieved in rural revitalization, including urbanization, aging population, rural population loss, industrialization, agricultural modernization and rural digitalization [23]. The study of rural entrepreneurial response subsystems focuses on the behaviors and resource elements of multiple subjects in the rural entrepreneurial community, including rural entrepreneurs and employees (labor force), government (policy and land), banks (finance), colleges/universities and research institutions (innovation and technology) [24,25]. With the state of rural entrepreneurship as the dependent variable and rural entrepreneurship pressure and response as the independent variables, we quantitatively measured the impacts of different factors using geographically weighted regression models and Geodetector, and empirically analyzed the theoretical hypotheses of spatial and composite effects of rural entrepreneurship. Based on the analysis results, rural entrepreneurship management strategies were proposed, that is, spatial zoning and spatial alliance strategies for rural entrepreneurship spatial planning according to the heterogeneity and autocorrelation of rural entrepreneurship space; and a policy classification and combination model for rural entrepreneurship management based on the nature, intensity, and interaction of factor effects. The design and implementation of management strategies feed back into the rural entrepreneurial response subsystem, guiding multiple subjects to change their behavior and factor allocation plans, further contributing to the adjustment of the rural entrepreneurial state subsystem, and ultimately transmitting and counteracting in the rural entrepreneurial context to promote elevation, release, or maintenance of stability of pressure (Fig 1).

The technical route and key analytical steps of the empirical study are as follows: The first step was to construct the evaluation indicator system of rural entrepreneurship index, and quantitatively measure the rural entrepreneurship index and its growth rate of 59 counties in Anhui based on official statistics and big data crawled from the Internet. The second step is to construct an indicator system for analyzing the influencing factors of rural entrepreneurship based on the theory of rural entrepreneurship pressure and response subsystems, and collect relevant data. The third step was to quantitatively analyze the changing trend, geographical distribution pattern and spatial effect of rural entrepreneurship in 59 counties of Anhui using python and GIS. The spatial heterogeneity test was performed using the coefficient of variation and quantile spatial clustering analysis methods, while the spatial autocorrelation test was performed using Moran’s index and cold/hot spot spatial analysis methods. The third step was to quantitatively analyze the nature, intensity and spatial difference of the influence of different factors on rural entrepreneurship using a geographically weighted regression model. The fourth step was to quantitatively analyze the interaction between different factors through the geographic detector software, and empirically test the composite effect of factors. The fifth step is to conduct management strategy research based on the analysis results, carry out superposition analysis on spatial and composite effects of rural entrepreneurship, and propose countermeasures and suggestions for rural entrepreneurship spatial planning and management policy design.

### Research methods and data sources

#### Spatial econometric model.

(1) Spatial Heterogeneity: Coefficient of Variation and Spatial Clustering

The coefficient of variation (CV) is a classic data heterogeneity analysis method used in statistics, and a larger value represents a larger difference between rural entrepreneurship indexes in different counties of Anhui. According to the study of Zhao [26], spatial heterogeneity was categorized into three levels, that is, high, medium and low, with thresholds of 0.16 and 0.36. Based on the analysis of coefficient of variation, the spatial heterogeneity of rural entrepreneurship in Anhui was visualized using the quantile spatial cluster analysis method of GIS, and the rural entrepreneurship index was categorized into three levels: high, medium and low.

(2) Spatial Autocorrelation: Moran’s Index and Analysis of Cold and Hot Spots

Moran’s index and analysis of hot and cold spots are the most commonly used methods for measuring global spatial autocorrelation and visualizing local spatial autocorrelation, and in this paper, they were used to quantitatively detect spatial correlation of rural entrepreneurship [27]. A positive Moran’s index represents a positive spatial autocorrelation in the geographic distribution pattern of rural entrepreneurship, and vice versa. The hot and cold spot analysis requires the calculation of the $Getis-\;Ord\;G_{i}^{*}$ index, and it indicates that rural entrepreneurship is in a hot spot area when greater than 0, otherwise in a cold spot area. The rural entrepreneurship in 59 counties of Anhui was divided into four types: hot spot, cold spot, sub-hot spot and sub-cold spot, using the cold and hot spot analysis method. Moran’s index [28,29] and the $Getis-\;Ord\;G_{i}^{*}$ index [30] are calculated as follows:


$$\text{Global Moran}’\text{s }I=\frac{N}{S_{0}}\times \frac{\sum_{i=1}^{N}\sum_{j=1}^{N}W_{ij}(Y_{i}-\overset{―}{Y})(Y_{j}-\overset{―}{Y})}{\sum_{i=1}^{N}{\left(Y_{i}-\overset{―}{Y}\right)}^{2}},\;S_{0}=\sum_{i=1}^{N}\sum_{j=1}^{N}=W_{ij}$$ (1)

$$G_{i}^{*}(d)=\frac{\sum_{i=1}^{N}W_{ij}(d)y_{i}}{\sum_{i=1}^{N}y_{i}}$$ (2)

Where, N is the number of counties in Anhui Province; $Y_{i}$and $Y_{j}$ are the rural entrepreneurship indexes of counties i and j in Anhui Province, respectively; $\overset{―}{Y}$ is the average rural entrepreneurship index in Anhui Province; $W_{ij}$ is the spatial weight matrix (adjacency matrix); and $S_{0}$ is the sum of spatial weight matrices. In this paper, Moran’s Index was calculated and spatial cold and hot spots were analyzed using Arcgis10.2 and GeoDa1.18 based on the default parameters of the software.

(3) Spatial effect of factor action: Geographically Weighted Regression Model

Geographically weighted regression models include spatial effects of rural entrepreneurship in regression to provide more accurate analysis results than traditional statistical regression models while allowing for subsequent specialized analyses on a per-county basis [31]. In this study, the geographically weighted regression model was used to analyze the nature and degree of influence of different factors on the rural entrepreneurship index and its rate of change, and the regression coefficient of each county was calculated. The calculation equations are as follows [32]:


$$Y_{i}=\beta_{0_{\left(\mu_{i},\;v_{i}\right)}}+\sum_{k}\beta_{k_{\left(\mu_{i},\;v_{i}\right)}}X_{ik}+\epsilon_{i}$$ (3)

Where, $Y_{i}$ represents the rural entrepreneurship index of county i; $X_{ik}$ is the k-th influencing factor of county i in Anhui Province; $\beta_{\text{0}}$ is a constant term; $\left(\mu_{i},\;v_{i}\right)$ is the geographic center coordinate point of county i; $\beta_{k_{\left(\mu_{i},\;v_{i}\right)}}$ represents the correlation between the different influencing factors of county i; $\epsilon_{i}$ is the error term of the regression equation. Due to the high sensitivity of geographically weighted regression models to factor collinearity, so the VIF (variance inflation factor) parameter was calculated using a linear regression model. The calculation results showed that the VIFs of all 15 independent variables were less than 10, indicating weak collinearity among the indicators and meeting the requirements of the multiple regression model.

(4) Factor interaction relationship: Geodetector

Geodetector (http://www.geodetector.cn), created by Prof. Jinfeng Wang in China, is an emerging statistical method for analyzing the interaction between different factors [33,34]. Geodetector is a nonlinear spatial measurement model, and it was used in this study to quantitatively measure the interaction force of factor pairs acting together on rural entrepreneurship. In calculation Geodetector was first used to calculate the direct influence of the influencing factors $X_{i}$ and $X_{j}$ on rural entrepreneurship, represented by q ($X_{i}$) and q ($X_{j}$), then work out the interactive influence of the two factors together, represented by q ($X_{i}$∩$X_{j}$), and finally determine the interaction by a comparative analysis of the maximum, minimum, and sum values of the interaction force and the direct force. It is judged as non-linear Weaken when q ($X_{i}$∩$X_{j}$) <Min (q($X_{i}$), q($X_{j}$)); unitary non linear Weaken when Min (q($X_{i}$), q($X_{j}$)) < q ($X_{i}$∩$X_{j}$) <Max (q($X_{i}$)), q($X_{j}$)); bifactor enhancement when q($X_{i}$)+q($X_{j}$)>q ($X_{i}$∩$X_{j}$)> Max (q($X_{i}$), q($X_{j}$)); independent when q ($X_{i}$∩$X_{j}$)=q($X_{i}$)+q($X_{j}$); non-linear enhancement when q ($X_{i}$∩$X_{j}$) > q($X_{i}$)+q($X_{j}$) [35]. A larger value of q(index) indicates stronger interactive influence, with a maximum of 1. With h as the number of classification categories of the independent variables (which were divided into 2–11 layers by the quantile method and the maximum value that met the significance test was taken as the final calculation result in this study), $N_{h}$ as the number of counties within layer h, N = 59, $\sigma_{h}^{2}$ and $\sigma^{2}$ as the variances of layer h and the rural entrepreneurship index of Anhui, respectively, SSW as the sum of the variances within the layer, and SST as the total variance of the study area, q(index) is calculated as follows [36]:


$$\begin{array}{c} q(index)=1-\frac{\sum_{h=1}^{l}N_{h}\sigma_{h}^{2}}{N\sigma^{2}}=1-\frac{SSW}{SST},\;SSW=\sum_{h=1}^{l}N_{h}\sigma_{h}^{2},\;SST=\;N\sigma^{2} \end{array}$$ (4)

#### Indicator selection.

The dependent variable, i.e., the state indicator of rural entrepreneurship, is represented by the rural revitalization index and its growth rate. The independent variables are derived from the stress and response subsystem of rural entrepreneurship. Based on the studies of scholars such as Rado [37], Ivari [38], Nnodim [39], Nordbo [40], Romero-Castro [41], and Ghani [42], 15 indicators were selected in this paper, including Industrialization ($X_{\text{1}}$), Urbanization ($X_{\text{2}}$), Aging population ($X_{\text{3}}$), Population loss ($X_{\text{4}}$), Agricultural modernization ($X_{\text{5}}$), Rural Digitalization Index ($X_{\text{6}}$), Fiscal self-sufficiency rate ($X_{\text{7}}$), Agricultural financial expenditures ($X_{\text{8}}$), Rural industrial land area ($X_{\text{9}}$), Urban-rural income ratio ($X_{\text{10}}$), Average years of education ($X_{\text{11}}$), Per capita housing construction area ($X_{\text{12}}$), Average number of people per household ($X_{\text{13}}$), Financial institution loan balance ($X_{\text{14}}$), Rural Innovation Index ($X_{\text{15}}$) (Table 1), with Aging population ($X_{\text{3}}$), Population loss ($X_{\text{4}}$), and Urban-rural income ratio ($X_{\text{10}}$) being negative factors (Table 1).

**Table 1:** The influencing factors of rural entrepreneurship based on PSR model in Anhui province.

Dimension	Code	Variable	Calculation Method
Pressure	$X_{\text{1}}$	Industrialization	Per capita GDP: the gross domestic product divided by resident population.
	$X_{\text{2}}$	Urbanization	Urbanization rate of permanent population: the urban population divided by the total resident population.
	$X_{\text{3}}$	Aging population	Proportion of population aged 60 and above: the population over 60 divided by total population.
	$X_{\text{4}}$	Population loss	Proportion of population residing outside the province: the population whose permanent residence is outside Anhui Province divided by the total population whose permanent residence is not in registered residence.
	$X_{\text{5}}$	Agricultural modernization	Added value of primary industry: the value added of agriculture, forestry, animal husbandry, fisheries and related service industries.
	$X_{\text{6}}$	Rural digitalization	Digital village index
State	$Y_{\text{1}}$	Rural entrepreneurial strength	Rural entrepreneurship index
	$Y_{\text{2}}$	Rural entrepreneurial potential	Growth rate of rural entrepreneurship index
Response	$X_{\text{7}}$	Government financial sustainability	Fiscal self-sufficiency rate: the government revenue divided by expenditure.
	$X_{\text{8}}$	Government investment in agricultural industries	Agricultural financial expenditures: the government’s investment in agriculture, forestry, animal husbandry, fisheries, and related service industries.
	$X_{\text{9}}$	Rural industrial land resources supply	Rural industrial land area: the rural construction land used for manufacturing, commerce, logistics and other industries
	$X_{\text{10}}$	Common prosperity of urban and rural	Urban-rural income ratio: the urban per capita income divided by rural per capita income.
	$X_{\text{11}}$	Education level of residents	Average years of education
	$X_{\text{12}}$	Household assets	Per capita housing construction area: the housing area divided by total population.
	$X_{\text{13}}$	Family situation	Average number of people per household: population divided by number of households
	$X_{\text{14}}$	Commercial bank financial support	Financial institution loan balance: the total bank loans minus repaid portion
	$X_{\text{15}}$	Rural innovation	Rural innovation index

#### Data sources.

The rural entrepreneurship data were derived from the China Rural Entrepreneurship Index Report (2023) jointly published by the China Rural Development Research Institute of Zhejiang University, the Zhejiang Sci-Tech University School of Economics and Management, and Qiyan Data Technology (Hangzhou) Co., Ltd., the first to cover rural entrepreneurship indexes of most provincial, municipal, and county-level administrative districts in China [43,44]. Among the independent variables, Urbanization, Aging population, Population loss, Education level of residents, Household assets, Family situation came from the Seventh National Population Census in 2020; Rural digitalization data were from the Research Report on County Digital Rural Index (2020) published by the Institute of New Rural Development of Peking University and Ali Research Institute [45]. Rural industrial land resources supply data were sourced from the Third National Land Resources Survey, while the rest data were from the Anhui Provincial Statistical Yearbook, with missing data from the statistical bulletins of counties and cities in Anhui.

## Results

### Spatial characteristics

#### Spatial clustering.

Jinzhai had the highest rural entrepreneurship index in 2014 at 25.71; Linquan had the lowest at 5.55. In 2021, Sixian had the highest rural entrepreneurship index at 37.07; Wuwei had the lowest at 8.90. The maximum was 4.63 times the minimum in 2014 and narrowed to 4.17 times in 2021, which was still significant although the gap between them was gradually closing. The coefficient of variation in 2014 and 2021 were 0.31 and 0.37, respectively, indicating significant spatial heterogeneity in the geographical distribution of the rural entrepreneurship index in Anhui with grade shifting from medium to high, showing an upward trend. The differences between different counties should not be ignored in the management of rural entrepreneurship in Anhui. The rural entrepreneurship index in 2014 and 2021, and the growth rate of rural entrepreneurship index from 2014 to 2021 were categorized into high, medium, and low levels by quantile spatial cluster analysis method.

The geographic distribution of rural entrepreneurship in 2014 showed the following characteristics. Firstly, high-value regions included Jinzhai, Hanshan, Shitai, Yuexi, Feixi, Sixian, Guangde, Langxi, Huoshan, Jingde, Dingyuan, Tianchang, Dangtu, Qimen, Dangshan, Feidong, Taihu and Fengtai, which were highly dispersed and formed a small belt-shaped agglomeration only in the western part of Anhui. Secondly, the median-value regions included Wangjiang, Hexian, Jixi, Mengcheng, Wuwei, Qingyang, Lingbi, Susong, Jieshou, Changfeng, Suixi, Tongcheng, Nanling, Xiaoxian, Dongzhi, Xiuning and Lixin, which formed two cluster-like agglomerations in the southwest and northeast of the province. Thirdly, low-value regions included Guzhen, Huaining, Chaohu, Jingxian, Lujiang, Laian, Fengyang, Wuhe, Zongyang, Shexian, Mingguang, Shucheng, Huaiyuan, Huoqiu, Funan, Yingshang, Shouxian and Linquan, which formed three belt-shaped agglomerations in central Hefei-Lu’an, eastern Bengbu-Chuzhou, and western Bengbu-Huainan (Table 2).

**Table 2:** The spatial clustering of rural entrepreneurship index and its growth rate in Anhui province.

Type	Rural Entrepreneurship Index in 2014	Rural Entrepreneurship Index in 2021	Growth Rate of Rural Entrepreneurship Index from 2014 to 2021
High	Jinzhai, Hanshan, Shitai, Yuexi, Feixi, Sixian, Guangde, Langxi, Huoshan, Jingde, Dingyuan, Tianchang, Dangtu, Qimen, Dangshan, Feidong, Taihu, Fengtai, Guoyang	Sixian, Changfeng, Xiaoxian, Lixin, Mengcheng, Huoqiu, Yingshang, Guzhen, Jinzhai, Nanling, Jieshou, Yuexi, Feixi, Feidong, Guoyang, Fengtai, Lingbi, Wuhe, Dingyuan	Yingshang, Huoqiu, Lixin, Xiaoxian, Changfeng, Guzhen, Wuhe, Linquan, Sixian, Funan, Nanling, Mengcheng, Zongyang, Jieshou, Shucheng, Shouxian, Lingbi, Huaiyuan, Huaining
Medium	Qianshan, Yixian, Wangjiang, Hexian, Jixi, Mengcheng, Wuwei, Qingyang, Lingbi, Susong, Jieshou, Changfeng, Suixi, Tongcheng, Nanling, Xiaoxian, Dongzhi, Xiuning, Lixin, Ningguo	Huoshan, Zongyang, Dangshan, Shitai, Tianchang, Yixian, Jingde, Huaining, Guangde, Suixi, Shucheng, Susong, Taihe, Taihu, Qingyang, Funan, Qianshan, Huaiyuan, Dongzhi, Langxi	Feidong, Guoyang, Fengtai, Taihe, Suixi, Dingyuan, Chaohu, Mingguang, Dangshan, Feixi, Yixian, Dongzhi, Huoshan, Lujiang, Yuexi, Susong, Fengyang, Tianchang, Jingxian, Qingyang
Low	Quanjiao, Taihe, Guzhen, Huaining, Chaohu, Jingxian, Lujiang, Laian, Fengyang, Wuhe, Zongyang, Shexian, Mingguang, Shucheng, Huaiyuan, Huoqiu, Funan, Yingshang, Shouxian, Linquan	Wangjiang, Chaohu, Hexian, Linquan, Dangtu, Xiuning, Lujiang, Quanjiao, Qimen, Mingguang, Ningguo, Jingxian, Jixi, Fengyang, Shouxian, Laian, Shexian, Tongcheng, Hanshan, Wuwei	Quanjiao, Ningguo, Shexian, Xiuning, Laian, Jingde, Taihu, Qianshan, Wangjiang, Guangde, Jinzhai, Hexian, Shitai, Tongcheng, Jixi, Dangtu, Qimen, Langxi, Wuwei, Hanshan

The geographical distribution of rural entrepreneurship in 2021 is as follows. First, the high-value regions included Sixian, Changfeng, Xiaoxian, Lixin, Mengcheng, Huoqiu, Yingshang, Guzhen, Jinzhai, Nanling, Jieshou, Yuexi, Feixi and Feidong, with emergence of three clusters, namely Bozhou-Huainan-Fuyang-Lu’an junction in the north, the southeast of Suzhou, and the Chuzhou-Hefei junction. Second, the median-value regions included Dangshan, Shitai, Tianchang, Yixian, Jingde, Huaining, Guangde, Suixi, Shucheng, Susong, Taihe, Taihu, Qingyang and Funan, with most of the agglomerations distributed in the southwest of Anhui Province. Third, low-value regions included Linquan, Dangtu, Xiuning, Lujiang, Quanjiao, Qimen, Mingguang, Ningguo, Jingxian, Jixi, Fengyang, Shouxian, Laian, Shexian, Tongcheng, Hanshan and Wuwei, with most of the agglomerations located in the periphery of the provincial capital metropolitan area.

For the geographical distribution of changes in rural entrepreneurship from 2014 to 2021, first, the growth rate of all high-value regions was more than 6%, mostly over 10%, with Yingshang and Huoqiu reaching 20%. Yingshang, Huoqiu, Lixin, Xiaoxian, Changfeng, Guzhen, Wuhe, Linquan, Sixian, Funan, Nanling, Mengcheng, Zongyang, and Jieshou were high-value areas and they were distributed in northern Anhui in a belt shape. Second, the growth rates of the median-value regions were all above 1% but less than 6%. Fengtai, Taihe, Suixi, Dingyuan, Chaohu, Mingguang, Dangshan, Feixi, Yixian, Dongzhi, Huoshan, Lujiang, Yuexi, Susong and Fengyang median-value regions, and they were distributed in the central part of Anhui in the form of a band. Third, the low-value regions were clustered in the south of Anhui and extended to the center along the Yangtze River and the provincial border. Most of the low-value regions had negative growth rates, geographically covering Qianshan, Wangjiang, Guangde, Jinzhai, Hexian, Shitai, Tongcheng, Jixi, Dangtu, Qimen, Langxi, Wuwei and Hanshan; and a small number of them had slow growth rates of less than 1%, covering geographical areas such as Xiuning, Laian, Jingde and Taihu.

#### Spatial effect.

The Moran’s index of rural entrepreneurship index in 2014 was 0.13 (P < 0.05, Z = 1.80), indicating a significant positive spatial autocorrelation in rural entrepreneurship in Anhui. Hot spots and cold spots were clustered in geographical distribution, with the former forming 4 small agglomerations and the latter forming 2 agglomerations. The agglomerations of the hot spots were the eastern region of Chuzhou City, the Lu’an-Anqing junction area in western Anhui, the Huangshan-Chizhou junction area in the southwest, and the Hefei-Chuzhou-Ma’anshan junction area in the east. The cold spot agglomerations were the Fuyang-Hainan-Lu’an junction in western Anhui and the Hefei-Anqing-Tongling junction area in the central part. The sub-hot and sub-cold spots were on the periphery of the hot and cold spots and extended in a band to the periphery (Table 3).

**Table 3:** The spatial effect of rural entrepreneurship index and its growth rate in Anhui province.

City	Rural Entrepreneurship Index in 2014	Rural Entrepreneurship Index in 2021	Growth Rate of Rural Entrepreneurship Index from 2014 to 2021
Chaohu	Sub-cold spot	Hot spot	Hot spot
Changfeng	Sub-cold spot	Hot spot	Sub-hot spot
Feidong	Sub-hot spot	Sub-hot spot	Sub-hot spot
Feixi	Cold spot	Cold spot	Sub-cold spot
Lujiang	Hot spot	Cold spot	Cold spot
Suixi	Sub-cold spot	Sub-hot spot	Sub-hot spot
Guoyang	Sub-cold spot	Cold spot	Sub-cold spot
Mengcheng	Sub-cold spot	Hot spot	Hot spot
Lixin	Sub-cold spot	Hot spot	Hot spot
Dangshan	Cold spot	Hot spot	Hot spot
Xiaoxian	Sub-cold spot	Hot spot	Hot spot
Lingbi	Cold spot	Sub-hot spot	Hot spot
Sixian	Sub-hot spot	Sub-cold spot	Cold spot
Huaiyuan	Sub-hot spot	Cold spot	Cold spot
Wuhe	Hot spot	Cold spot	Cold spot
Guzhen	Sub-hot spot	Hot spot	Sub-hot spot
Jieshou	Cold spot	Sub-cold spot	Sub-cold spot
Linquan	Sub-hot spot	Cold spot	Sub-cold spot
Taihe	Hot spot	Sub-hot spot	Sub-cold spot
Funan	Sub-hot spot	Sub-cold spot	Sub-cold spot
Yingshang	Sub-cold spot	Sub-cold spot	Sub-cold spot
Fengtai	Hot spot	Sub-hot spot	Sub-cold spot
Shouxian	Cold spot	Cold spot	Sub-hot spot
Tianchang	Hot spot	Sub-cold spot	Sub-cold spot
Mingguang	Sub-cold spot	Cold spot	Sub-cold spot
Laian	Sub-hot spot	Sub-cold spot	Sub-cold spot
Quanjiao	Hot spot	Sub-cold spot	Cold spot
Dingyuan	Hot spot	Sub-cold spot	Cold spot
Fengyang	Sub-hot spot	Sub-cold spot	Sub-cold spot
Huoqiu	Hot spot	Cold spot	Cold spot
Shucheng	Sub-hot spot	Sub-hot spot	Sub-cold spot
Jinzhai	Sub-cold spot	Sub-hot spot	Sub-hot spot
Huoshan	Sub-hot spot	Sub-cold spot	Sub-cold spot
Dangtu	Cold spot	Cold spot	Sub-cold spot
Hanshan	Cold spot	Sub-hot spot	Hot spot
Hexian	Sub-cold spot	Sub-hot spot	Sub-hot spot
Wuwei	Cold spot	Sub-cold spot	Hot spot
Nanling	Cold spot	Hot spot	Hot spot
Ningguo	Cold spot	Sub-hot spot	Hot spot
Guangde	Sub-hot spot	Hot spot	Sub-hot spot
Langxi	Sub-hot spot	Hot spot	Sub-hot spot
Jingxian	Sub-hot spot	Hot spot	Hot spot
Jixi	Sub-hot spot	Hot spot	Hot spot
Jingde	Cold spot	Hot spot	Hot spot
Zongyang	Sub-cold spot	Sub-cold spot	Sub-cold spot
Dongzhi	Hot spot	Hot spot	Sub-cold spot
Shitai	Hot spot	Hot spot	Sub-cold spot
Qingyang	Sub-cold spot	Hot spot	Sub-hot spot
Tongcheng	Sub-cold spot	Hot spot	Hot spot
Qianshan	Sub-hot spot	Hot spot	Hot spot
Huaining	Sub-hot spot	Sub-cold spot	Sub-cold spot
Taihu	Hot spot	Sub-cold spot	Cold spot
Susong	Sub-cold spot	Sub-hot spot	Sub-hot spot
Wangjiang	Hot spot	Sub-cold spot	Cold spot
Yuexi	Sub-hot spot	Sub-hot spot	Sub-cold spot
Shexian	Sub-cold spot	Cold spot	Cold spot
Xiuning	Sub-hot spot	Cold spot	Cold spot
Yixian	Sub-cold spot	Cold spot	Sub-cold spot
Qimen	Hot spot	Sub-cold spot	Cold spot

The Moran’s index for rural entrepreneurship in 2021 was 0.24 (P < 0.003, Z = 3.09), indicating a further improved spatial positive autocorrelation of rural entrepreneurship in Anhui. Hot and cold spots changed from clustering to banding in geographical distribution, and they began to take the shape of a “center-periphery” structure in spatial pattern. The hot spot cluster zone was located in northern Anhui, covering a number of prefecture-level cities, including Bozhou, Suzhou, Huaibei, Huainan, Bengbu and Lu’an. Most of the cold spots were clustered in the provincial capital metropolitan area (Anqing-Hefei-Ma’anshan), with a small number in the Huangshan and Huangshan Junction area in the southern part. The sub-hot spots were scattered in the fringe areas of the hot spots, and the sub-cold spots were relatively concentrated in the Huangshan-Chizhou-Anqing junction area in the southwest of the province.

The Moran’s index of the growth rate from 2014 to 2021 was 0.46 (P < 0.001, Z = 5.62), indicating the highest level of spatial positive autocorrelation of the growth rate of rural entrepreneurship index in Anhui. The spatial clustering system of the geographical distribution of hot and cold spots took shape, and the “center-periphery” structure of the spatial pattern was well-developed. The hot spots were distributed in a continuous strip along the Huaihe River, all concentrated in northern Anhui. The cold spots formed four cluster-like agglomerations of different sizes, all located in the south of Anhui, including the Hefei-Ma’anshan-Chuzhou junction area, the Huangshan-Anqing junction area, and the eastern and western ends of Xuancheng. Most of the sub-hot spots were distributed in the periphery of the hot spots, relatively concentrated in the area north of the Huaihe River, including Huaibei and Suzhou. The sub-cold spots were in the south of Huaihe River, mostly concentrated in the fringe areas of southwestern and southern Anhui, with a few in Chuzhou City.

### Spatial attribution

#### Rural entrepreneurship index.

Table 4 provides a statistical analysis of the geographically weighted regression coefficients of the 15 factors, and the results show that different factors have significant differences in the nature and intensity of their effects on the rural entrepreneurship index in Anhui. According to the absolute value of mean and median of geographically weighted regression coefficients, the intensity of the effect of the influencing factors was classified into key, important and auxiliary levels. Aging population ($X_{\text{3}}$) and average number of people per household ($X_{\text{13}}$) have much higher influence on rural entrepreneurship in Anhui, and they are key influencing factors. Rural innovation index ($X_{\text{15}}$), financial institution loan balance ($X_{\text{14}}$), urban-rural income ratio ($X_{\text{10}}$), industrialization ($X_{\text{1}}$), per capita housing construction area ($X_{\text{12}}$), average years of education ($X_{\text{11}}$), fiscal self-sufficiency rate ($X_{\text{7}}$), agricultural financial expenditures ($X_{\text{8}}$) also have a great influence on rural entrepreneurship in Anhui, and they are important influencing factors. Other factors have a weak influence on rural entrepreneurship in Anhui, especially the rural industrial land area ($X_{\text{9}}$) with the weakest influence, and they are auxiliary influencing factors. Based on the changes in the geographically weighted regression coefficients min, upper-quartile, median, lower-quartile, and max, the nature of the effect of the influence factors was classified into three categories: positive facilitating, negative inhibiting and mixed. The rural digitalization index ($X_{\text{6}}$), the average years of education ($X_{\text{11}}$), and the per capita housing construction area ($X_{\text{12}}$) are positive facilitating factors, and the improvement of their development level can drive the rise of Anhui rural entrepreneurship index, so they should be the focus in policy design. Urbanization ($X_{\text{2}}$), aging population ($X_{\text{3}}$), fiscal self-sufficiency rate ($X_{\text{7}}$), urban-rural income ratio ($X_{\text{10}}$), average number of people per household ($X_{\text{13}}$), financial institution loan balance ($X_{\text{14}}$), rural innovation index ($X_{\text{15}}$) are negative inhibiting factors, and they will make rural entrepreneurship in Anhui increasingly difficult. They should be dealt with in policy design by the following three strategies: avoiding as much as possible; controlling their status through policy design to reduce their negative inhibiting force on rural entrepreneurship; and designing policy combinations based on factor interactions to promote a flip in the nature or intensity of their effects. Industrialization ($X_{\text{1}}$), population loss ($X_{\text{4}}$), gricultural modernization ($X_{\text{5}}$), agricultural financial expenditures ($X_{\text{8}}$), and rural industrial land area ($X_{\text{9}}$) are mixed factors, and their effects will reverse in different regions and have an extremely complex influence mechanism on rural entrepreneurship, so special research is needed in policy design.

**Table 4:** Statistical analysis of geographically weighted regression coefficients for rural entrepreneurship index in Anhui province.

Variable	Mean	Min	Upper-quartile	Median	Lower-quartile	Max
$X_{\text{1}}$	−1.72	−2.87	−2.32	−1.95	−1.30	1.52
$X_{\text{2}}$	−0.83	−1.51	−1.11	−0.83	−0.62	−0.08
$X_{\text{3}}$	−3.52	−3.84	−3.72	−3.59	−3.38	−2.94
$X_{\text{4}}$	−0.33	−1.14	−0.64	−0.32	−0.03	0.47
$X_{\text{5}}$	0.54	−1.01	0.00	0.61	1.14	2.15
$X_{\text{6}}$	0.59	0.28	0.53	0.62	0.65	0.72
$X_{\text{7}}$	1.10	0.34	0.78	1.00	1.40	2.26
$X_{\text{8}}$	1.15	−0.58	0.24	0.92	1.97	3.95
$X_{\text{9}}$	0.15	−0.80	−0.06	0.14	0.39	0.84
$X_{\text{10}}$	−1.55	−2.07	−1.72	−1.56	−1.36	−1.17
$X_{\text{11}}$	1.72	1.09	1.38	1.65	2.00	2.98
$X_{\text{12}}$	1.69	0.33	0.91	1.44	2.29	4.77
$X_{\text{13}}$	−2.45	−3.24	−2.75	−2.45	−2.20	−1.72
$X_{\text{14}}$	−1.56	−3.76	−2.13	−1.38	−0.93	−0.42
$X_{\text{15}}$	−1.78	−3.57	−2.20	−1.68	−1.29	−0.90

The mechanism of influencing factors showed a significant spatial effect, and there were large spatial differences in the influence of different factors on rural entrepreneurship in Anhui. Industrialization ($X_{\text{1}}$) exerted a strong negative inhibiting effect on rural entrepreneurship in the southwest of the province, contributing to a belt shaped agglomeration area that extended eastward along the central and southern parts of Anhui. It played a strong positive facilitating role in the north, but covered a very small geographical area. Urbanization ($X_{\text{2}}$) played a negative inhibiting effect on all rural entrepreneurship in Anhui, with the strong inhibitory area clustered in the southwest and the weak inhibitory area clustered in the northeast, forming a spatial pattern of gradient decline from southwest to northeast. Aging population ($X_{\text{3}}$) played a negative inhibiting effect on all rural entrepreneurship in Anhui, with the strong inhibitory area concentrated in the north, and the weak inhibitory areas concentrated in the south, forming a spatial pattern of gradient decline from the north to the south. Population loss ($X_{\text{4}}$) played both positive facilitating and negative inhibiting effects on rural entrepreneurship, with strong negative inhibiting areas concentrated at the western edge of the province, strong positively acting areas concentrated at the eastern edge, and the influence of the factor gradually decreasing from the east and west to the central part, forming a U-shaped spatial pattern high in the east and west and concave in the center. Agricultural modernization ($X_{\text{5}}$) had a mixed effect on rural entrepreneurship, with a spatial pattern very similar to that of population loss ($X_{\text{4}}$). The factors rural digitalization index ($X_{\text{6}}$) and fiscal self-sufficiency rate ($X_{\text{7}}$) played a positive role in promoting all rural entrepreneurship, and the spatial pattern of their effects was characterized by gradient and great difference in spatial structure of force changes, with the former high in the south and low in the north, while the latter high in the west and low in the east. Agricultural financial expenditures ($X_{\text{8}}$) had a mixed influence on rural entrepreneurship, and the spatial pattern of its force was characterized by a high north and a low south, with a positive facilitating effect in most areas and a negative inhibiting effect only in the southern fringe areas. The rural industrial land area ($X_{\text{9}}$) has a mixed influence on rural entrepreneurship in Anhui, with the high value positively acting area clustered in the southwest and the high value negatively acting area in the northeast, forming a U-shaped spatial pattern high in the southwest and northeast and concave in the middle. Urban-rural income ratio ($X_{\text{10}}$) exerted a negative inhibiting effect on all rural entrepreneurship in the province, with the high-value areas clustered in the north, especially in the northeast, and the geological acting areas in the south, forming a spatial pattern of gradient changes with high north and low south. Average years of education ($X_{\text{11}}$) and per capita housing construction area ($X_{\text{12}}$) both served as positive promoters for rural entrepreneurship in Anhui, with the high-value areas clustered in the north and the low-value areas in the south, forming a spatial pattern of gradient changes with north high and south low. Average number of people per household ($X_{\text{13}}$), financial institution loan balance ($X_{\text{14}}$) and rural innovation index ($X_{\text{15}}$) all had a negative inhibitory effect on rural entrepreneurship in Anhui, with a highly similar spatial pattern of the force, except for some differences only in some areas. The high-value areas were clustered in the north and the low-value areas in the south, forming a spatial pattern of gradient changes with north high and south low (Table 5).

**Table 5:** The geographical weighted regression analysis results of rural entrepreneurship index in Anhui province.

City	$X_{1}$	$X_{2}$	$X_{3}$	$X_{4}$	$X_{5}$	$X_{6}$	$X_{7}$	$X_{8}$	$X_{9}$	$X_{10}$	$X_{11}$	$X_{12}$	$X_{13}$	$X_{14}$	$X_{15}$
Yingshang	−1.48	−0.80	−3.78	−0.64	0.05	0.55	1.46	1.89	0.28	−1.65	1.96	2.18	−2.83	−2.08	−2.08
Huoqiu	−1.80	−0.93	−3.77	−0.68	−0.15	0.58	1.55	1.58	0.43	−1.57	1.82	1.86	−2.77	−1.84	−1.88
Lixin	−0.99	−0.66	−3.77	−0.69	0.22	0.50	1.43	2.36	0.11	−1.73	2.17	2.70	−2.95	−2.47	−2.37
Xiaoxian	0.72	−0.12	−3.64	−0.45	1.18	0.29	0.59	3.48	−0.68	−2.03	2.75	4.09	−3.08	−3.28	−3.23
Changfeng	−1.70	−0.76	−3.68	−0.29	0.48	0.58	1.08	1.41	0.15	−1.64	1.82	1.79	−2.52	−1.69	−1.91
Guzhen	−0.87	−0.39	−3.81	−0.16	0.96	0.45	0.79	2.26	−0.24	−1.86	2.18	2.64	−2.72	−2.31	−2.45
Wuhe	−1.00	−0.34	−3.78	0.01	1.19	0.47	0.66	2.07	−0.29	−1.86	2.11	2.50	−2.61	−2.16	−2.38
Linquan	−1.36	−0.88	−3.67	−1.14	−0.42	0.61	2.26	2.34	0.53	−1.57	2.07	2.74	−3.20	−2.62	−2.23
Sixian	−0.58	−0.14	−3.84	0.10	1.43	0.39	0.47	2.44	−0.49	−1.96	2.28	2.90	−2.70	−2.43	−2.65
Funan	−1.57	−0.91	−3.78	−0.92	−0.33	0.57	1.89	2.00	0.49	−1.58	1.96	2.30	−3.01	−2.24	−2.06
Nanling	−2.18	−0.91	−3.26	−0.03	1.00	0.68	0.85	0.29	0.11	−1.43	1.44	1.06	−2.02	−0.98	−1.39
Mengcheng	−0.92	−0.56	−3.80	−0.49	0.48	0.48	1.16	2.33	−0.03	−1.78	2.18	2.67	−2.86	−2.40	−2.40
Zongyang	−2.32	−1.09	−3.43	−0.35	0.22	0.66	1.17	0.47	0.37	−1.41	1.45	0.98	−2.24	−1.01	−1.37
Jieshou	−0.91	−0.76	−3.61	−1.08	−0.15	0.57	2.05	2.64	0.32	−1.66	2.24	3.10	−3.20	−2.85	−2.46
Shucheng	−2.23	−1.07	−3.59	−0.49	−0.05	0.63	1.35	0.84	0.45	−1.47	1.56	1.22	−2.45	−1.26	−1.52
Shouxian	−1.73	−0.83	−3.72	−0.45	0.21	0.57	1.26	1.49	0.26	−1.62	1.83	1.82	−2.63	−1.75	−1.91
Lingbi	−0.38	−0.17	−3.84	−0.05	1.27	0.37	0.55	2.66	−0.48	−1.97	2.36	3.09	−2.80	−2.59	−2.74
Huaiyuan	−1.10	−0.54	−3.79	−0.31	0.66	0.49	1.00	2.09	−0.06	−1.78	2.09	2.44	−2.73	−2.19	−2.30
Huaining	−2.54	−1.27	−3.50	−0.58	−0.34	0.65	1.44	0.39	0.59	−1.34	1.36	0.80	−2.36	−0.90	−1.24
Feidong	−1.80	−0.76	−3.60	−0.18	0.67	0.60	0.97	1.19	0.11	−1.61	1.75	1.65	−2.40	−1.54	−1.82
Guoyang	−0.45	−0.51	−3.72	−0.68	0.47	0.45	1.28	2.78	−0.11	−1.82	2.37	3.21	−3.03	−2.80	−2.65
Fengtai	−1.35	−0.71	−3.78	−0.50	0.29	0.53	1.26	1.94	0.15	−1.70	2.01	2.25	−2.77	−2.10	−2.15
Taihe	−0.74	−0.68	−3.64	−0.96	0.05	0.53	1.78	2.69	0.17	−1.72	2.29	3.14	−3.14	−2.84	−2.52
Suixi	−0.36	−0.38	−3.78	−0.45	0.77	0.41	0.96	2.77	−0.27	−1.89	2.39	3.18	−2.94	−2.73	−2.71
Dingyuan	−1.52	−0.60	−3.67	−0.11	0.86	0.55	0.87	1.51	−0.03	−1.70	1.88	1.95	−2.48	−1.77	−2.02
Chaohu	−1.95	−0.83	−3.52	−0.16	0.69	0.63	0.97	0.92	0.14	−1.56	1.65	1.44	−2.30	−1.36	−1.68
Mingguang	−1.26	−0.38	−3.70	0.12	1.32	0.51	0.62	1.72	−0.25	−1.80	1.98	2.23	−2.47	−1.93	−2.20
Dangshan	1.52	−0.08	−3.39	−0.71	1.16	0.28	0.53	3.95	−0.80	−2.07	2.98	4.77	−3.24	−3.76	−3.57
Feixi	−2.02	−0.94	−3.62	−0.40	0.19	0.61	1.23	1.06	0.32	−1.54	1.66	1.44	−2.47	−1.43	−1.68
Yixian	−2.45	−1.23	−3.15	−0.25	0.50	0.69	1.02	−0.23	0.29	−1.25	1.20	0.53	−1.94	−0.58	−1.05
Dongzhi	−2.66	−1.39	−3.37	−0.56	−0.33	0.65	1.38	0.01	0.57	−1.25	1.22	0.49	−2.22	−0.62	−1.03
Huoshan	−2.37	−1.18	−3.69	−0.71	−0.49	0.63	1.63	0.91	0.64	−1.43	1.54	1.20	−2.62	−1.29	−1.48
Lujiang	−2.17	−1.00	−3.51	−0.32	0.30	0.64	1.15	0.74	0.31	−1.48	1.56	1.21	−2.32	−1.20	−1.53
Yuexi	−2.56	−1.28	−3.65	−0.75	−0.66	0.63	1.69	0.67	0.73	−1.36	1.43	0.97	−2.58	−1.09	−1.32
Susong	−2.87	−1.51	−3.58	−0.83	−1.01	0.62	1.75	0.18	0.84	−1.22	1.20	0.50	−2.50	−0.67	−0.99
Fengyang	−1.30	−0.51	−3.73	−0.10	0.93	0.52	0.82	1.78	−0.11	−1.77	1.99	2.19	−2.56	−1.96	−2.17
Tianchang	−1.30	−0.15	−3.60	0.47	2.03	0.52	0.34	1.52	−0.45	−1.85	1.95	2.28	−2.31	−1.86	−2.21
Jingxian	−2.25	−0.97	−3.18	−0.02	1.04	0.70	0.84	0.05	0.12	−1.37	1.35	0.90	−1.93	−0.84	−1.27
Qingyang	−2.32	−1.06	−3.28	−0.19	0.61	0.68	1.00	0.18	0.25	−1.37	1.37	0.87	−2.05	−0.86	−1.28
Quanjiao	−1.72	−0.63	−3.55	0.02	1.07	0.60	0.78	1.15	−0.04	−1.65	1.76	1.71	−2.31	−1.54	−1.85
Ningguo	−2.18	−0.85	−3.01	0.19	1.68	0.72	0.63	−0.25	−0.05	−1.34	1.28	0.91	−1.74	−0.78	−1.23
Shexian	−2.32	−1.14	−2.94	−0.04	1.16	0.71	0.78	−0.58	0.10	−1.20	1.12	0.48	−1.72	−0.51	−0.99
Xiuning	−2.44	−1.31	−3.02	−0.24	0.61	0.68	0.95	−0.54	0.25	−1.17	1.09	0.33	−1.84	−0.42	−0.90
Laian	−1.47	−0.39	−3.58	0.24	1.54	0.56	0.56	1.38	−0.25	−1.75	1.87	2.02	−2.32	−1.73	−2.05
Jingde	−2.29	−1.03	−3.08	−0.01	1.12	0.71	0.81	−0.20	0.11	−1.31	1.26	0.75	−1.84	−0.71	−1.16
Taihu	−2.75	−1.40	−3.63	−0.81	−0.88	0.63	1.75	0.43	0.81	−1.29	1.31	0.73	−2.55	−0.88	−1.15
Qianshan	−2.53	−1.26	−3.57	−0.64	−0.45	0.64	1.53	0.53	0.63	−1.36	1.41	0.90	−2.45	−1.00	−1.29
Wangjiang	−2.68	−1.38	−3.48	−0.65	−0.54	0.64	1.51	0.20	0.66	−1.28	1.27	0.60	−2.35	−0.73	−1.09
Guangde	−2.06	−0.63	−3.04	0.38	2.15	0.71	0.49	−0.08	−0.19	−1.44	1.38	1.20	−1.74	−0.97	−1.37
Jinzhai	−2.40	−1.21	−3.78	−0.88	−0.76	0.62	1.90	1.10	0.78	−1.41	1.57	1.34	−2.80	−1.45	−1.52
Hexian	−1.85	−0.68	−3.45	0.06	1.17	0.63	0.76	0.89	−0.02	−1.59	1.67	1.55	−2.20	−1.38	−1.73
Shitai	−2.49	−1.24	−3.27	−0.36	0.19	0.68	1.15	0.00	0.39	−1.29	1.26	0.62	−2.08	−0.68	−1.12
Tongcheng	−2.35	−1.13	−3.53	−0.48	−0.07	0.65	1.33	0.62	0.47	−1.42	1.48	1.04	−2.37	−1.09	−1.41
Jixi	−2.28	−1.03	−3.00	0.03	1.29	0.72	0.75	−0.39	0.06	−1.27	1.20	0.67	−1.75	−0.63	−1.10
Dangtu	−1.95	−0.68	−3.34	0.15	1.39	0.66	0.69	0.62	−0.06	−1.54	1.58	1.41	−2.07	−1.24	−1.61
Qimen	−2.54	−1.33	−3.19	−0.38	0.17	0.67	1.14	−0.24	0.39	−1.22	1.17	0.43	−2.01	−0.53	−0.98
Langxi	−2.03	−0.63	−3.14	0.33	1.93	0.69	0.54	0.17	−0.16	−1.48	1.46	1.28	−1.84	−1.06	−1.46
Wuwei	−2.11	−0.92	−3.43	−0.16	0.65	0.65	0.98	0.64	0.19	−1.49	1.55	1.22	−2.21	−1.17	−1.53
Hanshan	−1.92	−0.76	−3.47	−0.04	0.94	0.63	0.85	0.87	0.06	−1.57	1.65	1.47	−2.23	−1.35	−1.69

There was a significant synergistic enhancement effect between different factors, with most factor pairs in the non-linear enhancement relationship and a few in the bifactor enhancement relationship. Only 16 out of 105 factor pairs are in the bifactor enhancement relationship, including industrialization fiscal ∩ self-sufficiency rate ($X_{\text{1}}\;\cap \;X_{\text{7}}$), industrialization ∩ agricultural financial expenditures ($X_{\text{1}}$ ∩ $X_{\text{8}}$), urbanization ∩ agricultural modernization ($X_{\text{2}}$ ∩ $X_{\text{5}}$), urbanization ∩ fiscal self-sufficiency rate ($X_{\text{2}}$ ∩ $X_{\text{7}}$), urbanization ∩ average number of people per household ($X_{\text{2}}$ ∩ $X_{\text{13}}$), urbanization ∩ financial institution loan balance ($X_{\text{2}}$ ∩ $X_{\text{14}}$), and urbanization ∩ rural innovation index ($X_{\text{2}}$ ∩ $X_{\text{15}}$), mostly composed of urbanization ($X_{\text{2}}$), fiscal self-sufficiency rate ($X_{\text{7}}$), urban-rural income ratio ($X_{\text{10}}$), average number of people per household ($X_{\text{13}}$) and other factors. The interaction of factor pairs varied greatly, with a maximum value of 0.95 and a minimum value of only 0.09, a difference of more than 10 times between the two. The interaction forces of factor pairs such as population loss ∩ agricultural financial expenditures ($X_{\text{4}}$ ∩ $X_{\text{8}}$), population loss ∩ financial institution loan balance ($X_{\text{4}}$ ∩ $X_{\text{14}}$), population loss ∩ rural innovation index ($X_{\text{4}}$ ∩ $X_{\text{15}}$), financial institution loan balance ∩ rural innovation index ($X_{\text{14}}$ ∩ $X_{\text{15}}$), population loss ∩ aging population ($X_{\text{4}}$ ∩ $X_{\text{3}}$), aging population ∩ rural innovation index ($X_{\text{3}}$ ∩ $X_{\text{15}}$) were more than 0.9, more than twice the influence of their independent action, and they were super factor pairs. The factor pairs including fiscal self-sufficiency rate ∩ urban-rural income ratio ($X_{\text{7}}$ ∩ $X_{\text{10}}$), fiscal self-sufficiency rate ∩ rural digitalization index ($X_{\text{7}}$ ∩ $X_{\text{6}}$), fiscal self-sufficiency rate ∩ rural industrial land area ($X_{\text{7}}$ ∩ $X_{\text{9}}$), fiscal self-sufficiency rate ∩ average number of people per household ($X_{\text{7}}$ ∩ $X_{\text{13}}$), urban-rural income ratio ∩ average number of people per household ($X_{\text{10}}$ ∩ $X_{\text{13}}$) generally had an interaction force less than 0.20, indicating weak synergistic effects (Table 6).

**Table 6:** The interaction relationship and effects of factors for rural entrepreneurship index in Anhui province.

Variable	$X_{1}$	$X_{2}$	$X_{3}$	$X_{4}$	$X_{5}$	$X_{6}$	$X_{7}$	$X_{8}$	$X_{9}$	$X_{10}$	$X_{11}$	$X_{12}$	$X_{13}$	$X_{14}$	$X_{15}$
$X_{\text{1}}$	0.23														
$X_{\text{2}}$	0.38	0.22													
$X_{\text{3}}$	0.78	0.62	0.36												
$X_{\text{4}}$	0.67	0.59	0.93	0.24											
$X_{\text{5}}$	0.73	*0.49*	*0.64*	0.77	0.31										
$X_{\text{6}}$	0.48	0.49	0.71	0.48	0.50	0.04									
$X_{\text{7}}$	*0.28*	*0.26*	0.55	0.45	0.43	0.20	0.03								
$X_{\text{8}}$	*0.62*	0.53	0.80	0.95	0.69	0.67	0.34	0.25							
$X_{\text{9}}$	0.52	0.40	0.55	0.59	0.48	0.26	0.16	0.41	0.10						
$X_{\text{10}}$	0.32	*0.29*	0.48	0.40	*0.37*	0.28	*0.09*	*0.31*	*0.18*	0.05					
$X_{\text{11}}$	0.64	0.60	0.87	0.84	0.88	0.53	0.45	0.84	0.52	0.30	0.14				
$X_{\text{12}}$	0.63	0.53	0.75	0.79	0.65	0.48	0.21	0.63	0.49	0.28	0.63	0.11			
$X_{\text{13}}$	0.44	*0.30*	0.53	0.41	0.51	0.21	*0.16*	0.41	*0.26*	*0.18*	0.60	0.36	0.13		
$X_{\text{14}}$	0.72	*0.70*	0.83	0.94	0.72	0.74	0.45	0.74	0.43	0.44	0.70	0.91	0.57	0.25	
$X_{\text{15}}$	0.76	*0.67*	0.92	0.95	0.88	0.66	0.60	0.85	*0.63*	0.57	0.76	0.88	0.63	0.95	0.45

Note: The italics and red represent the bifactor enhancement relationship, while others represent the non-linear enhancement relationship. The diagonal data represents the influence of factors acting independently.

#### Growth rate of rural entrepreneurship index.

Table 4 provides a statistical analysis of the geographically weighted regression coefficients of the 15 factors, and the results show that different factors have significant differences in the nature and intensity of their effects on the rural entrepreneurship index growth rate in Anhui. According to the absolute value of mean and median of geographically weighted regression coefficients, the intensity of the effect of the influencing factors was classified into key, important and auxiliary levels. Urbanization ($X_{\text{2}}$), aging population ($X_{\text{3}}$), average number of people per household ($X_{\text{13}}$), and rural innovation index ($X_{\text{15}}$) have a much higher influence on the change of rural entrepreneurship in Anhui than other factors, and they are key influencing factors. Agricultural modernization ($X_{\text{5}}$), rural digitization index ($X_{\text{6}}$), and per capital housing construction area ($X_{\text{12}}$) also have a strong influence on the changes in rural entrepreneurship in Anhui, and they are important influencing factors. Other factors have a weak influence on rural entrepreneurship in Anhui, especially the agricultural financial expenditures ($X_{\text{8}}$) with the weakest influence, and they are auxiliary influencing factors. According to the changes in the geographically weighted regression coefficients min, upper-quartile, median, lower-quartile, and max, it was found that the mechanisms of the 15 factors influencing the changes in rural entrepreneurship in Anhui were very complex, and all of them were mixed factors with none appearing to play an only positive facilitating or negative inhibiting role (Table 7).

**Table 7:** Statistical analysis of geographically weighted regression coefficients for growth rate of rural entrepreneurship index in Anhui province.

Variable	Mean	Min	Upper-quartile	Median	Lower-quartile	Max
$X_{\text{1}}$	−0.47	−9.81	−2.59	−1.39	2.21	8.25
$X_{\text{2}}$	−1.94	−10.58	−3.83	−2.11	−0.89	11.02
$X_{\text{3}}$	2.16	−10.64	−1.89	0.54	4.49	18.73
$X_{\text{4}}$	−0.23	−12.48	−2.32	−0.22	2.03	10.25
$X_{\text{5}}$	1.35	−18.38	−0.97	1.58	3.97	24.81
$X_{\text{6}}$	1.13	−8.27	−0.35	1.70	2.42	10.75
$X_{\text{7}}$	0.53	−8.28	−1.71	1.25	2.69	7.91
$X_{\text{8}}$	0.08	−9.61	−1.91	−0.05	2.10	14.73
$X_{\text{9}}$	0.58	−3.83	−0.43	0.28	1.19	5.96
$X_{\text{10}}$	−0.31	−6.79	−2.92	−0.51	2.17	12.44
$X_{\text{11}}$	0.11	−10.86	−1.69	0.83	2.47	9.52
$X_{\text{12}}$	1.59	−3.89	0.26	1.67	2.93	12.71
$X_{\text{13}}$	−1.94	−13.78	−4.59	−2.38	0.34	8.72
$X_{\text{14}}$	0.64	−20.48	−1.28	−0.38	3.17	12.42
$X_{\text{15}}$	−2.02	−10.68	−2.92	−1.41	−0.52	9.27

The influencing factors have more significant spatial effect on the change of rural entrepreneurship in Anhui, and their effects generally exhibit clustering characteristics, but the spatial pattern of influence of different factors is completely different. Most of the positive strong acting areas of industrialization ($X_{\text{1}}$) were clustered in the northern part of Anhui, with a small portion in the southwest, and the negative strong acting clusters were in the west and southeast, respectively. Most of the positively acting areas of urbanization ($X_{\text{2}}$) were clustered in the central province, while the negatively acting areas formed two agglomerations in the northeast and southwest, with a greater influence in the fringe areas. Aging population ($X_{\text{3}}$) played a positive role in most areas, and formed a large high-value center in the north of Anhui; the negatively acting areas formed two clusters in the southeast and west. Population loss ($X_{\text{4}}$) had a positive facilitating effect on most areas and formed a small high-value center in the north; the negatively acting areas formed a large agglomeration in the southwest. Agricultural modernization ($X_{\text{5}}$) played a positive role in most areas, and formed three high-value centers in central, eastern and southeastern Anhui, and a small negative agglomeration area in the southwest. The positive concentration areas of rural digitalization index ($X_{\text{6}}$) covered most of central and northern Anhui, and formed two positive high-value centers in the north and west; the negatively acting areas were clustered in the southern fringe. Most of the positively acting areas of fiscal self-sufficiency rate ($X_{\text{7}}$) were concentrated in central Anhui, and formed a large high-value center in the west and a small high-value center in the southwest. Most of the negatively acting areas were concentrated in the north of Anhui, and a small portion was concentrated in the southwest. The spatial pattern of the influence of agricultural financial expenditures ($X_{\text{8}}$) was opposite to that of rural digitalization index ($X_{\text{6}}$), with the positive high-value centers located in the south of Anhui and the negative high-value centers in the north. Rural industrial land area ($X_{\text{9}}$) played a negative role for most of the area, with the high-value centers located in the provincial capital metropolitan area and the eastern part of Xuancheng. Most of the positively acting areas were clustered in the west and north of Anhui, and the high-value centers were in a continuous distribution along the western edge of the province. The positively acting areas of urban-rural income ratio ($X_{\text{10}}$) were clustered in the north and the negatively acting areas were in the south, without forming a distinct high-value center. The positive and negatively acting areas of average years of education ($X_{\text{11}}$) accounted for half of Anhui, and the interaction changed from negative to positive from northwest to southeast, with the high-value centers in the fringe at the two ends of northwest and southeast. Per capita housing construction area ($X_{\text{12}}$) had a positive influence on most areas, forming two high-value centers in the provincial capital metropolitan area and Xuancheng. The negatively acting area covered a very small geographical area, forming a small agglomeration only in the northeast of Anhui. Average number of people per household ($X_{\text{13}}$) had a negative inhibitory effect on most areas and formed two high-value centers, one large and one small, in Anqing and Xuancheng. Most of the positive areas were located in the north of Anhui and formed a large high-value center. The positively and negatively acting areas of financial institution loan balance ($X_{\text{14}}$) each covered half of the geographical area of Anhui, with the positive high-value center located in the north and both negative high-value centers in the south. Rural innovation index ($X_{\text{15}}$) had a negative inhibitory effect on most regions, with the center of the high negative values in the north of Anhui and the sub-center in the central part. The positively acting areas were distributed in the southwest of Anhui, with no mature positive high-value center formed (Table 8).

**Table 8:** The geographical weighted regression analysis results of growth rate of rural entrepreneurship index in Anhui province.

City	$X_{1}$	$X_{2}$	$X_{3}$	$X_{4}$	$X_{5}$	$X_{6}$	$X_{7}$	$X_{8}$	$X_{9}$	$X_{10}$	$X_{11}$	$X_{12}$	$X_{13}$	$X_{14}$	$X_{15}$
Yingshang	0.28	−0.20	2.37	3.75	3.63	1.66	2.25	−0.05	1.27	2.42	−2.59	1.40	−0.97	−0.46	−1.68
Huoqiu	−2.79	−0.25	−0.15	3.01	3.22	1.49	4.15	−0.25	2.18	3.33	−2.20	0.84	−2.28	−0.83	0.10
Lixin	4.07	−2.28	9.50	5.48	0.13	2.89	−2.93	−3.33	1.76	−0.28	−4.62	−0.79	3.14	5.49	−5.87
Xiaoxian	2.77	0.08	10.75	6.82	3.63	0.66	−1.87	−4.73	0.74	3.82	−4.45	1.08	5.27	2.61	−6.32
Changfeng	−1.80	−2.10	2.47	0.64	4.71	2.29	1.97	0.26	0.47	1.08	1.53	3.36	−1.90	−0.28	−2.03
Guzhen	4.68	−5.26	8.29	0.20	3.39	3.08	−2.73	−2.39	0.51	2.17	−0.92	0.12	2.26	5.54	−3.94
Wuhe	3.36	−4.61	5.11	−1.11	4.34	2.53	−1.54	−1.93	0.10	3.42	−0.12	0.86	0.50	3.13	−1.16
Linquan	−5.78	9.75	8.65	3.12	−1.10	10.75	−3.33	−5.35	5.96	−6.76	−10.86	−3.89	4.66	−5.37	−10.68
Sixian	4.60	−4.67	4.75	−0.99	3.40	2.22	−3.49	−1.96	−0.35	2.50	−0.06	0.43	0.66	3.78	−1.87
Funan	−0.16	−0.72	0.22	1.87	0.72	2.25	3.97	1.90	3.29	1.54	−6.32	2.97	−3.07	−1.37	0.06
Nanling	−1.24	−1.07	−2.25	0.67	3.52	0.42	1.48	2.78	−1.50	−2.36	1.91	2.98	−4.63	−1.66	−1.39
Mengcheng	6.82	−4.79	11.40	5.23	−0.07	2.94	−5.06	−3.37	0.71	−0.72	−3.10	−0.50	4.04	8.29	−6.68
Zongyang	0.88	−2.87	0.54	−2.26	0.60	1.24	0.31	3.14	−0.92	−1.82	1.52	1.71	−4.55	−1.28	−0.59
Jieshou	−6.93	−2.40	8.35	10.25	−4.02	−8.27	4.60	−5.12	3.11	12.44	−5.35	−2.37	8.72	5.12	−0.07
Shucheng	−8.20	−0.04	−0.47	−0.52	1.58	2.45	5.30	−0.31	0.91	0.33	0.09	1.63	−3.04	0.56	−1.33
Shouxian	−2.30	−1.15	1.17	1.64	5.18	1.98	2.28	−0.33	0.83	2.50	0.33	2.26	−2.55	−0.49	−1.20
Lingbi	7.26	−7.29	10.27	−0.52	1.63	3.36	−5.94	−3.25	0.20	1.61	−0.79	−0.39	3.38	7.75	−4.07
Huaiyuan	4.23	−4.76	8.20	1.94	3.21	2.92	−1.95	−1.87	0.64	1.41	−1.24	0.26	2.27	5.53	−4.94
Huaining	−0.48	−3.64	0.61	−4.40	−1.36	1.06	1.15	1.17	−0.10	−1.67	1.45	1.67	−6.03	−0.01	−0.52
Feidong	−2.35	−2.11	2.71	0.55	4.19	2.22	2.40	0.96	0.08	−0.51	2.45	3.95	−1.39	−0.38	−2.64
Guoyang	7.43	−5.35	15.71	3.04	−2.21	3.73	−7.26	−6.47	3.14	−1.37	−5.26	−1.28	4.71	8.47	−6.54
Fengtai	1.94	−1.19	4.43	4.40	3.84	1.92	0.73	−0.61	0.44	2.18	−1.98	1.38	0.17	1.40	−2.93
Taihe	4.16	−6.06	17.43	3.26	−5.07	1.01	−5.94	−9.61	5.52	2.28	−6.67	−3.71	7.07	10.08	−4.38
Suixi	5.15	−4.18	10.81	4.97	0.97	3.26	−4.13	−3.69	1.13	0.58	−3.57	−0.96	4.17	6.73	−6.51
Dingyuan	0.11	−3.63	3.58	0.02	4.34	2.39	1.12	−0.21	0.28	1.22	1.41	2.75	−1.20	0.95	−2.28
Chaohu	−2.51	−1.27	1.47	0.68	3.90	1.78	2.06	1.73	−0.56	−1.31	2.42	3.71	−1.12	−0.61	−2.31
Mingguang	1.75	−3.25	2.10	−0.62	4.05	1.95	−0.52	−0.73	−0.09	2.41	0.28	1.35	−0.54	1.35	−0.82
Dangshan	5.64	3.78	18.73	2.26	2.49	−0.41	−5.64	−9.51	3.61	2.59	−4.98	−0.28	6.97	4.82	−7.90
Feixi	−7.01	0.09	0.30	0.99	3.40	2.36	4.17	−0.05	0.69	0.21	1.00	2.40	−2.22	−0.15	−1.63
Yixian	−1.51	−2.71	−2.99	−4.02	−6.77	−1.79	2.92	7.42	−0.45	−3.20	4.22	−0.42	−3.45	−3.79	0.44
Dongzhi	−1.39	−3.61	0.11	−5.63	−3.39	−0.78	−1.03	−0.23	−0.01	−3.67	2.61	1.74	−5.39	1.78	−0.07
Huoshan	−9.22	−0.65	−2.03	−4.04	1.06	2.70	6.22	−1.88	2.34	2.28	−0.94	0.70	−5.26	1.05	−1.39
Lujiang	−2.64	−1.52	1.12	−0.58	2.34	1.80	2.43	2.14	−0.38	−1.22	1.67	2.70	−1.99	−0.78	−1.38
Yuexi	−7.52	−2.41	−2.41	−5.02	0.24	2.57	7.91	−1.49	2.36	1.69	−0.76	0.43	−6.78	−1.19	−1.69
Susong	8.25	−10.58	−2.43	−12.48	−2.84	−2.29	−8.28	−5.89	−2.45	−5.03	0.59	6.86	−13.78	8.59	2.17
Fengyang	1.70	−4.02	4.54	−0.22	4.38	2.53	−0.07	−1.13	0.23	2.50	0.25	1.34	0.02	2.63	−2.13
Tianchang	6.40	−6.93	1.20	−4.20	−5.58	1.38	3.71	3.53	−0.69	7.61	−7.25	2.10	0.83	12.42	−7.58
Jingxian	−2.11	−1.55	−2.76	−0.53	1.04	−0.29	2.18	2.91	−0.53	−2.79	2.50	2.52	−4.49	−1.79	−0.91
Qingyang	−1.67	−1.99	−1.79	−1.26	−0.36	−0.01	1.81	3.12	−0.41	−2.19	1.85	2.04	−4.22	−1.09	−0.52
Quanjiao	−1.50	−2.51	1.38	0.75	4.51	1.70	1.47	0.33	−0.05	−0.93	2.68	3.32	−2.58	−0.38	−2.91
Ningguo	−4.05	−0.68	−4.01	−0.52	4.96	−1.55	2.64	3.81	0.20	−3.81	6.32	3.65	−2.88	−6.34	−0.09
Shexian	−2.10	6.44	4.25	−3.09	−18.38	−2.89	−0.20	2.05	0.06	−4.56	−5.94	0.26	−6.14	10.39	9.27
Xiuning	1.28	−1.86	−2.15	−4.41	−7.82	−1.90	1.68	9.62	−0.66	−3.27	4.25	−2.63	−1.64	−5.64	0.86
Laian	−0.13	−4.85	0.86	−0.24	4.14	1.77	1.25	−1.11	0.79	−0.10	2.81	3.99	−4.49	−0.14	−2.81
Jingde	−2.97	−1.84	−2.84	−1.57	−0.83	−1.03	2.75	2.86	0.44	−3.06	3.15	1.90	−3.84	−2.33	−0.18
Taihu	−3.20	−4.42	−2.93	−5.20	−0.27	3.33	5.81	−1.38	2.02	0.49	−1.40	2.24	−8.90	−1.25	−0.82
Qianshan	−4.02	−2.74	−0.05	−3.32	−0.28	2.19	4.34	0.32	0.98	−0.34	0.74	0.47	−6.04	−1.28	−1.06
Wangjiang	1.38	−5.55	0.02	−7.52	−3.10	−0.94	−2.23	−1.32	−0.83	−3.53	2.17	3.26	−7.89	3.22	0.22
Guangde	−4.04	11.02	−10.64	4.87	24.81	−2.66	−3.17	14.73	−3.83	−6.79	9.52	12.71	−6.96	−20.48	−1.56
Jinzhai	−9.81	0.20	−0.79	−2.37	1.52	2.64	5.49	−3.59	5.79	4.29	0.98	−3.17	−1.91	−0.52	−1.31
Hexian	−1.80	−1.50	−0.45	1.27	4.73	1.11	1.19	0.87	−0.62	−2.05	3.06	3.27	−3.25	−0.93	−3.05
Shitai	0.28	−3.44	−1.46	−4.93	−3.83	−0.58	−0.19	2.53	0.31	−3.25	2.63	0.86	−5.47	−1.43	−0.20
Tongcheng	−2.54	−2.25	0.52	−2.10	0.55	1.88	2.77	1.69	0.03	−0.74	0.83	1.68	−4.55	−0.52	−0.85
Jixi	−3.50	−1.98	−2.22	−2.27	−2.47	−1.75	3.27	1.82	1.60	−3.36	2.45	1.70	−3.88	−2.05	0.91
Dangtu	−1.19	−0.49	−3.20	2.13	6.33	0.50	0.65	1.64	−1.93	−3.30	3.62	3.76	−5.43	−2.16	−3.46
Qimen	2.47	−3.51	−1.98	−7.00	−5.77	−0.91	−1.55	2.89	1.25	−4.33	3.18	−0.82	−5.03	−4.01	−0.51
Langxi	−2.14	5.46	−6.76	4.19	16.25	−1.00	−0.85	7.81	−3.81	−5.47	6.41	8.30	−6.73	−10.98	−2.02
Wuwei	−1.74	−1.20	−0.04	0.48	3.69	1.27	1.40	2.37	−1.11	−1.61	2.08	2.90	−2.38	−1.29	−1.41
Hanshan	−2.02	−1.40	0.42	1.07	4.31	1.40	1.44	1.30	−0.64	−1.70	2.62	3.25	−2.34	−0.69	−2.74

For the growth rate of rural entrepreneurship index: there was also a significant synergistic enhancement effect between different factors, and the number of factor pairs in the non-linear enhancement relationship and the bifactor enhancement relationship was almost equal. Factor pairs such as rural digitalization index ($X_{\text{6}}$), average years of education ($X_{\text{11}}$), per capita housing construction area ($X_{\text{12}}$) were mostly in the non-linear enhancement relationship; and those including population loss ($X_{\text{4}}$), fiscal self-sufficiency rate ($X_{\text{7}}$), agricultural financial expenditures ($X_{\text{8}}$), rural industrial land area ($X_{\text{9}}$) were in the bifactor enhancement relationship. The interaction of factor pairs varied greatly, with a maximum value of 0.99 and a minimum value of only 0.12, a difference also closes to 10 times between the two. The interaction effect was overall weak compared to the rural entrepreneurship index. There were only two factor pairs with an interaction force greater than 0.9, Including financial institution loan balance ∩ rural innovation index ($X_{\text{14}}$ ∩ $X_{\text{15}}$) and urbanization ∩ agricultural modernization ($X_{\text{2}}$ ∩ $X_{\text{5}}$). Of note is that 16 factor pairs including agricultural financial expenditures ∩ rural innovation index ($X_{\text{8}}$ ∩ $X_{\text{15}}$), industrialization ∩ rural innovation index ($X_{\text{1}}$ ∩ $X_{\text{15}}$), industrialization ∩ financial institution loan balance ($X_{\text{1}}$ ∩ $X_{\text{14}}$), urbanization ∩ financial institution loan balance ($X_{\text{2}}$ ∩ $X_{\text{14}}$), agricultural modernization ∩ rural innovation index ($X_{\text{5}}$ ∩ $X_{\text{15}}$), agricultural modernization ∩ financial institution loan balance ($X_{\text{5}}$ ∩ $X_{\text{14}}$), agricultural financial expenditures ∩ financial institution loan balance ($X_{\text{8}}$ ∩ $X_{\text{14}}$), agricultural modernization ∩ per capita housing construction area ($X_{\text{5}}$ ∩ $X_{\text{12}}$) have an interaction force of 0.80 or more, and they are all super factor pairs. There were only two factor pairs with an interaction force below 0.20, Including rural digitalization index ∩ urban-rural income ratio ($X_{\text{6}}$ ∩ $X_{\text{10}}$), and fiscal self-sufficiency rate ∩ urban-rural income ratio ($X_{\text{7}}$ ∩ $X_{\text{10}}$) (Table 9).

**Table 9:** The interaction relationship and effects of factors for growth rate of rural entrepreneurship index in Anhui province.

Variable	$X_{1}$	$X_{2}$	$X_{3}$	$X_{4}$	$X_{5}$	$X_{6}$	$X_{7}$	$X_{8}$	$X_{9}$	$X_{10}$	$X_{11}$	$X_{12}$	$X_{13}$	$X_{14}$	$X_{15}$
$X_{\text{1}}$	0.45														
$X_{\text{2}}$	*0.80*	0.34													
$X_{\text{3}}$	*0.73*	*0.56*	0.27												
$X_{\text{4}}$	0.80	0.66	0.48	0.14											
$X_{\text{5}}$	*0.83*	0.94	0.79	*0.65*	0.49										
$X_{\text{6}}$	0.81	0.80	0.72	0.24	0.75	0.05									
$X_{\text{7}}$	*0.51*	*0.39*	*0.33*	*0.23*	0.63	0.21	0.08								
$X_{\text{8}}$	*0.83*	*0.73*	0.70	0.62	*0.77*	0.83	*0.51*	0.40							
$X_{\text{9}}$	*0.52*	0.52	*0.36*	*0.28*	*0.56*	*0.21*	0.31	*0.49*	0.13						
$X_{\text{10}}$	0.56	*0.41*	*0.32*	*0.21*	0.61	0.17	*0.12*	0.51	0.24	0.04					
$X_{\text{11}}$	0.76	0.58	0.60	0.39	0.73	0.43	0.33	0.79	*0.29*	0.26	0.14				
$X_{\text{12}}$	0.75	0.61	0.60	0.38	0.82	0.33	0.22	0.77	0.37	0.23	0.49	0.09			
$X_{\text{13}}$	0.73	0.64	*0.45*	*0.37*	*0.76*	0.46	*0.31*	0.74	*0.36*	0.35	0.58	0.56	0.25		
$X_{\text{14}}$	*0.86*	0.85	0.77	0.61	*0.81*	0.77	0.58	*0.77*	*0.50*	0.63	0.68	0.74	*0.69*	0.43	
$X_{\text{15}}$	*0.87*	*0.77*	0.86	0.77	*0.88*	0.71	0.57	0.89	*0.56*	*0.49*	0.80	0.60	0.73	0.99	0.43

Note: The italics and red represent the bifactor enhancement relationship, while others represent the non-linear enhancement relationship. The diagonal data represents the influence of factors acting independently.

### Spatial policy

#### Technical framework.

Management policies and spatial planning are playing a key role in the formation and evolution of rural entrepreneurship, and are programmatic documents guiding the synergistic participation of multiple interests in entrepreneurial activities [46]. In recent years, Anhui has introduced a series of rural entrepreneurship policies. For instance, the General Office of the Anhui Provincial Government issued the Implementation Opinions of the General Office of the People’s Government of Anhui Province on Supporting Migrant Workers and Other Groups in Returning to Their Hometowns for Entrepreneurship. Besides, Anhui Provincial Department of Human Resources and Social Security, the Development and Reform Commission, the Department of Finance, the Department of Natural Resources and other departments jointly released the Notice on Further Supporting and Promoting Entrepreneurship Among Migrant Workers and Other Returnees and the Notice of the General Office of the Anhui Provincial Government on Issuing the “Entrepreneurship in Jianghuai” Action Plan (2021–2025). The policy primarily focuses on financial support, tax incentives, entrepreneurship training, and carrier construction, forming a relatively comprehensive policy system. For example, returnees who start their first business and have been in business for more than one year are eligible for subsidies of up to 50,000 yuan. The province has recognized 178 provincial-level demonstration parks for migrant workers returning to entrepreneurship and 356 rural craftsmen masters, while piloting the construction of township-level talent stations in 50 counties (cities). However, the current rural entrepreneurship policies in Anhui are universal and general, failing to account for heterogeneity across different regions. Therefore, new policy designs need to integrate the evolutionary models and influencing factors of rural entrepreneurship, delineate geographical zones, and implement differentiated spatial management policies.

**Fig 2:**
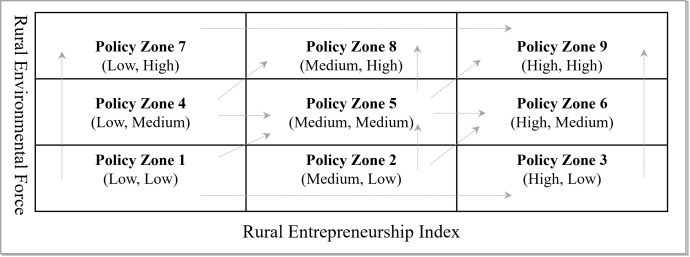
The technical framework for policy design based on overlay analysis in Anhui province.

Differentiated Management Spatial Policy design needs to incorporate the evolutionary patterns and influencing factors of rural entrepreneurship, delineate geographic zones and implement differentiated management policies. The technical framework and implementation steps of the policy design are as follows: weighting and summing the 15 influencing factors as the driving force for rural entrepreneurship, labeled as rural environmental force, with geographically weighted regression coefficients as weights [47]. A coordinate system is established using the rural entrepreneurship index and rural environmental force as horizontal and vertical coordinates. The two indicators are divided into three levels of low, medium and high by quantile spatial clustering, to create nine policy zones in the coordinate system. Adaptive management recommendations are made for each policy zone based on the policy zone of each county, as well as the spatial effects of rural entrepreneurship and the compounding effects of its influencing factors. Spatial zoning should take into account both the number and speed of rural entrepreneurship, which are the same in some areas, but more likely to have differences. The policy zones of counties are not fixed for a long time in the evolution of rural entrepreneurship, but are changed based on development dynamics and underlying conditions. Therefore, priority should be given to guiding them to evolve towards better policy zones in the direction as indicated by the arrow (Fig 2).

#### Spatial zoning.

The rural environmental force of 59 counties was classified into high, medium, and low levels by weighted summation and quantile spatial clustering using the geographically weighted regression coefficients of the rural entrepreneurship index and growth rate of rural entrepreneurship index, based on the standardized data of the impact factor indicators (Table 10). Apply the technical framework of Figure 7 to obtain the zoning plan for rural entrepreneurship policies in Anhui Province. The analysis results showed that 9 counties had the same spatial partition of rural entrepreneurship index and growth rate of rural entrepreneurship index, including Hanshan in policy zone 1, Taihe and Tianchang in policy zone 2, Yingshang in policy zone 3, Lingbi, Guzhen and Nanling in policy zone 6, Shexian in policy zone 7, and Huoqiu in policy zone 9. It is noteworthy that the results of the two were not the same in 85% of counties, so separate management is required for the quantity and speed of rural entrepreneurship.

**Table 10:** The interaction relationship and effects of factors for growth rate of rural entrepreneurship index in Anhui province.

Force	Rural Entrepreneurship Index	Growth Rate of Rural Entrepreneurship Index
High	Lixin, Dangshan, Xiaoxian, Shouxian, Huoqiu, Shucheng, Jinzhai, Huoshan, Hexian, Ningguo, Guangde, Langxi, Jingxian, Jixi, Jingde, Zongyang, Shitai, Qingyang, Qianshan, Shexian	Changfeng, Feidong, Feixi, Lujiang, Guoyang, Mengcheng, Sixian, Huaiyuan, Wuhe, Laian, Quanjiao, Huoqiu, Taihu, Susong, Wangjiang, Yuexi, Shexian, Xiuning, Yixian, Qimen
Medium	Guoyang, Mengcheng, Lingbi, Sixian, Wuhe, Guzhen, Fengtai, Mingguang, Quanjiao, Nanling, Dongzhi, Tongcheng, Huaining, Taihu, Susong, Wangjiang, Xiuning, Yixian, Qimen	Chaohu, Suixi, Lixin, Xiaoxian, Lingbi, Guzhen, Jieshou, Shouxian, Dingyuan, Fengyang, Shucheng, Jinzhai, Dangtu, Wuwei, Nanling, Jingde, Zongyang, Shitai, Huaining
Low	Chaohu, Changfeng, Feidong, Feixi, Lujiang, Suixi, Huaiyuan, Jieshou, Linquan, Taihe, Funan, Yingshang, Tianchang, Laian, Dingyuan, Fengyang, Dangtu, Hanshan, Wuwei, Yuexi	Dangshan, Linquan, Taihe, Funan, Yingshang, Fengtai, Tianchang, Mingguang, Huoshan, Hanshan, Hexian, Ningguo, Guangde, Langxi, Jingxian, Jixi, Dongzhi, Qingyang, Tongcheng, Qianshan

For the rural entrepreneurship index: Chaohu, Lujiang, Linquan, Laian, Fengyang, Dangtu, Hanshan and Wuwei are in policy zone 1, and it is difficult for them to change the overall backwardness of rural entrepreneurship simply by relying on their own strength due to their low level of rural entrepreneurship and lack of vitality. Suixi, Huaiyuan, Taihe, Funan, and Tianchang are in policy zone 2, with a moderate level of rural entrepreneurship but insufficient local motivation. Changfeng, Feidong, Feixi, Jieshou, Yingshang, Dingyuan, and Yuexi belong to policy zone 3, with significant internal obstacles even though their rural entrepreneurship was already at a high level. Mingguang, Quanjiao, Tongcheng, Wangjiang, Xiuning and Qimen are in policy zone 4, with a lower level of rural entrepreneurship despite their own favorable conditions. Dongzhi, Huaining, Taihu, Susong and Yixian are in policy zone 5, with moderate rural entrepreneurship at the middle level in Anhui. Guoyang, Mengcheng, Lingbi, Sixian, Wuhe, Guzhen, Fengtai and Nanling are in policy zone 6, with a high level of rural entrepreneurship and sufficient development momentum. Shouxian, Hexian, Ningguo, Jingxian, Jixi and Shexian are in policy zone 7, with a very strong rural entrepreneurial drive but a low level of development. Dangshan, Shucheng, Guangde, Langxi, Jingde, Zongyang, Shitai, Qingyang and Qianshan are in policy zone 8, with sufficient and high-level motivation, and they have a great potential to grow into leaders in rural entrepreneurship in the province in the future. Lixin, Xiaoxian, Huoqiu, Jinzhai and Huoshan are in policy zone 9, as star counties of rural entrepreneurship in Anhui (Table 11).

**Table 11:** The geospatial zoning for management policies of rural entrepreneurship in Anhui province.

Policy Zone	Quantity Management	Speed Management
Policy Zone 1	Chaohu, Lujiang, Linquan, Laian, Fengyang,Dangtu, Hanshan, Wuwei	Hanshan, Hexian, Ningguo, Guangde, Langxi, Jixi, Tongcheng, Qianshan
Policy Zone 2	Suixi, Huaiyuan, Taihe, Funan, Tianchang	Dangshan, Taihe, Fengtai, Tianchang, Mingguang, Huoshan, Jingxian, Dongzhi, Qingyang
Policy Zone 3	Changfeng, Feidong, Feixi, Jieshou, Yingshang, Dingyuan, Yuexi	Linquan, Funan, Yingshang
Policy Zone 4	Mingguang, Quanjiao, Tongcheng, Wangjiang, Xiuning, Qimen	Jinzhai, Dangtu, Wuwei, Jingde, Shitai
Policy Zone 5	Dongzhi, Huaining, Taihu, Susong, Yixian	Chaohu, Suixi, Dingyuan, Fengyang
Policy Zone 6	Guoyang, Mengcheng, Lingbi, Sixian, Wuhe, Guzhen, Fengtai, Nanling	Lixin, Xiaoxian, Lingbi, Guzhen, Jieshou, Shouxian, Shucheng, Nanling, Zongyang, Huaining
Policy Zone 7	Shouxian, Hexian, Ningguo, Jingxian, Jixi, Shexian	Laian, Quanjiao, Taihu, Wangjiang, Shexian, Xiuning, Qimen
Policy Zone 8	Dangshan, Shucheng, Guangde, Langxi, Jingde, Zongyang, Shitai, Qingyang, Qianshan	Feixi, Lujiang, Guoyang, Susong, Yuexi, Yixian
Policy Zone 9	Lixin, Xiaoxian, Huoqiu, Jinzhai, Huoshan	Changfeng, Feidong, Mengcheng, Sixian, Huaiyuan, Wuhe, Huoqiu

For the growth rate of rural entrepreneurship index: Hanshan, Hexian, Ningguo, Guangde, Langxi, Jixi, Tongcheng, Qianshan are in policy zone 1, with great internal development obstacles due to an extremely low or even negative growth rate. Dangshan, Taihe, Fengtai, Tianchang, Mingguang, Huoshan, Jingxian, Dongzhi and Qingyang are in policy zone 2, with a slow growth rate of rural entrepreneurship and insufficient incentive to change the status quo. Linquan, Funan, and Yingshang are in policy zone 3, with a rapid growth in rural entrepreneurship but a very weak impetus to maintain the growth, creating concerns for the future sustainability of rural entrepreneurship. Jinzhai, Dangtu, Wuwei, Jingde and Shitai are in policy zone 4, with a sufficient developmental momentum for rural entrepreneurship, which has not been translated effectively, resulting in a very low rate of development. Chaohu, Suixi, Dingyuan and Fengyang are in policy zone 5 with rural entrepreneurship development momentum and speed in the middle of Anhui. Lixin, Xiaoxian, Lingbi, Guzhen, Jieshou, Shouxian, Shucheng, Nanling, Zongyang and Huaining are in policy zone 6, with rural entrepreneurship development at a very high speed, but insufficient motivation to maintain the growth. Laian, Quanjiao, Taihu, Wangjiang, Shexian, Xiuning, and Qimen are in policy zone 7, with sufficient impetus for the development of rural entrepreneurship but a very low growth rate, indicating great problems in the transformation of impetus. Feixi, Lujiang, Guoyang, Susong, Yuexi, and Yixian are in policy zone 8, with a strong rural entrepreneurship momentum and a high growth rate. Changfeng, Feidong, Mengcheng, Sixian, Huaiyuan, Wuhe, and Huoqiu are in policy zone 9, with a leading edge in the growth rate and development momentum of rural entrepreneurship as star counties or star countries soon to be for rural entrepreneurship in Anhui.

For rural entrepreneurship management policy design: Provincial and municipal governments should increase direct investment and policy support for policy zone1 in the future, while encouraging them to establish development alliances with members of policy zone 9. Members of policy zone 1 can only activate their rural entrepreneurship by vigorously leveraging external forces in the future. Policy zone 1 can evolve into policy zones 2, 4, and 5 in the future based on the external forces and its own conditions. The core role of external forces is to directly intervene in the process of breaking the long-term backwardness. For policy zone 2, it is recommended to focus on optimizing the local rural entrepreneurial drive, identifying key obstacles that constrain rural entrepreneurship, promoting mechanism and system reforms, and unleashing rural entrepreneurial vitality. In addition, provincial and municipal governments can invest appropriately to leverage local resources to break the unfavorable situation of rural entrepreneurship as soon as possible. The core of moderate intervention by external forces lies in breaking the deadlock, not in policy support or spoiling by excessive enthusiasm. The selection of the evolution path for policy zone 2 needs to take into account the common characteristics of its own resources and external forces, while policy zone 5 is a less difficult choice to implement. Policy zone 3 should vigorously promote internal mechanism reform and development model innovation, break the current development bottleneck, and evolve into policy zone 6 as soon as possible in the future. The key to the policy design for policy zone 4 is to increase the integration of its own resources and the optimization of its development mechanism, so as to promote the transformation of development momentum into strength. For policy zone 5, the grassroots government and villagers should be encouraged to explore local development independently without unnecessary interference from the provincial and municipal governments. The future focus for policy zone 6 is to conduct specialized research on the internal influencing factors, investigate the key factors restricting development, and open up the choke point of local links of rural entrepreneurship early. It is suggested that the county government of policy zone 7 should invite a third-party institution to conduct a special study as soon as possible to deeply analyze the reasons for the failure of the transformation of rural entrepreneurial motivation and the path of error correction. When necessary, provincial and municipal governments may intervene appropriately to promote the removal of obstacles in the transformation of rural entrepreneurial motivation. It is recommended that provincial and municipal governments establish a cooperative mechanism with the county governments of policy zone 8 to make targeted investments in their weak areas and promote their evolution to policy zone 9. It is suggested that provincial and municipal governments choose members of policy zone 9 as demonstration or test sites to play a leading and innovative role internally, and participate in regional and national rural entrepreneurship competition externally. It is notable that in view of the fact that Anhui is still an underdeveloped region with limited financial resources, the government plays different roles in different policy zones [48]. Strong direct intervention is required in policy zone 1, and appropriate direct intervention is required in policy zone 2, policy zone 3, policy zone 4, and policy zone 7 to play a more supportive and guiding role there; in the rest policy areas, the government should only be moderately indirectly involved to play a more guiding and supporting role.

## Discussion

The construction of the theoretical system of rural entrepreneurship is still in its infancy. Most of the studies still focus on case studies and characterization summaries, generally done by qualitative or qualitative analytical methods. Empirical studies based on theoretical hypotheses are mainly in developed countries such as the United Kingdom, the United States, Spain, Finland and Greece, while there are few special studies on less developed countries, which is exactly the most potential and dynamic field of rural entrepreneurship research [49,50].

### Spatial effect

Based on the empirical analysis of Anhui, this study found that the spatial effect of rural entrepreneurship is mainly manifested in three areas: first, there are great differences in the status quo and change trend of rural entrepreneurship in different regions, with significant spatial heterogeneity. This law requires a zoning policy for rural entrepreneurship management, rather than a homogenized “one-size-fits-all” policy. Second, rural entrepreneurship in different regions has a significant positive spatial autocorrelation and model demonstration effect, as well as a proximity effect on regional flows of entrepreneurial resources. This law requires classified integration and overall coordination of resources in rural entrepreneurial management, focusing on supporting the establishment of development alliances in similar or adjacent regions to give play to regional synergies. Third, different factors have significant spatial heterogeneity in their impact on rural entrepreneurship, and the intensity and nature of their effects vary with geographical location. This law requires that the design of rural entrepreneurship management policies must take spatial effects into account, and failure to do so will result in the actual effects of policy implementation going against the expected goals.

Scholars have noticed the influence of geography and space in the study of rural entrepreneurship, and the marginal contribution of this study is the introduction of a spatial econometric modeling analytical approach, focusing on rural entrepreneurial activities. From the perspective of research methods, most scholars analyze the spatial pattern or geographical distribution characteristics of rural entrepreneurship through qualitative or qualitative methods such as case analysis, questionnaire survey and rooted analysis, but the quantitative empirical analysis is insufficient, and there is rare analysis using spatial econometric model [51]. Habersetzer found that local entrepreneurs get access to more opportunities than non-local entrepreneurs in the process of rural entrepreneurship in Sweden, and that local connections and experience have a positive impact on the survival and development of new enterprises [52]. Yu conducted an empirical study on the United States based on the joint model of location and entrepreneurial decision making, and found that rural entrepreneurship has a strong location preference. Instead of pursuing the location with the largest absolute economic return, it is more inclined to settle in the largest point of relative difference between urban entrepreneurship and rural entrepreneurship [53]. Muñoz [54] and Korsgaard [55] constructed a research framework for place-bound rural entrepreneurship based on socio-spatial theory. Müller found in case analysis of 28 rural entrepreneurial enterprises that spatial context plays a role in resource endowment and connecting bridge in rural entrepreneurial activities [56]. In terms of research objects, most scholars focus their research on holistic entrepreneurial activities or on the spatial effects of urban entrepreneurship, manufacturing entrepreneurship, service entrepreneurship, high-tech entrepreneurship and other popular areas, hardly with specialized research on rural entrepreneurship [57,58]. Hong analyzed the spatio-temporal dynamics of entrepreneurship and its determinants in Korea and concluded that the geographic distribution of entrepreneurship is spatially heterogeneous, time-dependent, and geographically correlated [59]. According to the spatio-temporal analysis of economic and census data, Zheng argued that the distribution of entrepreneurship in China’s manufacturing and service sectors is uneven, and that the emergence and evolution of entrepreneurial space clusters are closely related to city size, population age structure, and foreign direct investment [60].

It is important to note that the Implementing Opinions on Supporting the Return of Migrant Workers and Others to Rural Entrepreneurship and the Implementing Opinions on Promoting the High-Quality Development of Entrepreneurship in Returning to Rural Areas currently promulgated and implemented in Anhui have made no response to the spatial effects of rural entrepreneurship, although they clarify the main objectives and key tasks of rural entrepreneurship, the supply of elements and policy support, the service system, and the organizational safeguards. Therefore, it is recommended that Anhui revise its rural entrepreneurship management policies as soon as possible, incorporate spatial effects, emphasize differentiation and linkage between different regions, and formulate rural entrepreneurship spatial planning and resource allocation policies early. In addition, currently only a few county governments such as those of Shucheng and Zongyang have developed local rural entrepreneurship implementation plans, action plans, and path research reports. On the contrary, most counties have directly adopted the generic policies of provincial and municipal governments without formulating their own rural entrepreneurship management policies, which has led to problems such as insufficiently targeted and inadequate policy support. Therefore, provincial and municipal governments are recommended to support, guide, and help county governments to make their own rural entrepreneurship management policies or spatial planning for rural entrepreneurship governance with laws and expectations.

### Composite effect

**Fig 3:**
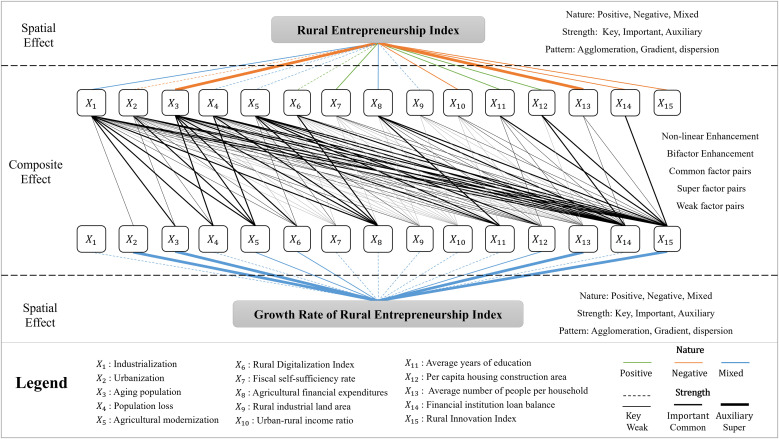
The summary of empirical analysis results on theoretical hypotheses.

Rural entrepreneurship is the result of multi-factor interaction and the academic consensus, but the mechanism of interaction has long been a gray box in academic research. This study found that although there are large differences in the influence of different factors on rural entrepreneurship, the existing interaction effects may bring changes to the nature and intensity of the effect of factors. This study found that according to the intensity of influence, factors can be classified as key, important, and auxiliary factors; in addition, the common effect of different factors on rural entrepreneurship is manifested as a synergistic enhancement effect, mainly shown as non-linear enhancement relationship and bifactor enhancement relationship, with emergence of a large number of super factor pairs. The influence of super factor pairs on rural entrepreneurship tends to be more than two times the separate influence of a single factor, or up to about 10 times in some cases. Thus, non-key single factors (auxiliary factors) acting separately are likely to play a critical role in the whole with through interaction effects. It is especially noteworthy that the effects of factors are not stable in nature across space, and the superposition of interaction and spatial effects is often likely to contribute to the transformation of a cofactor that originally played a positive facilitating role into a negative inhibiting key factor when multiple factors act together (Fig 3).

The analysis results of this study have something in common with the findings of other scholars, but are not identical or even opposite in some areas. The commonality is manifested in the fact factors such as rural innovation, entrepreneurs’ education level, urbanization and rural digitalization have great influence on rural entrepreneurship, which is the field to be focused on in policy design and government management [61]. In incomplete or even opposite fields: Römer-Paakkanen found in a study on over 50 entrepreneurs that as the old population has accumulated more developed networks, stronger financial situations, greater leverage resources, and the ability to create more trustworthy businesses, they are better suited than the younger generation to start new and more successful businesses [62]. In short, age is a motivational factor for rural entrepreneurship, but this study found it is not true in the overall analysis at the macro level. For some regions, aging has a negative inhibitory effect on the status quo of rural entrepreneurial activities, and has both positive and negative effects on their evolution, with great differences in performance in different regions. It fully proves the viewpoint mentioned above that the overall law of rural entrepreneurship is not equal to the simple sum of individual laws, and that the holistic law can only be truly revealed through a large or full sample study. The inhibitory effect of aging on rural entrepreneurship manifests as the absence of rural entrepreneurship subjects and insufficient employment population and labor supply, reflecting the structural dependence of traditional rural economies on labor. The promotive effect of aging on rural entrepreneurship is evident in the emergence of new sectors and demands, such as elderly care services and intergenerational care, driven by the silver economy. Meanwhile, the success of rural entrepreneurship relies on localized resources and social capital, with older entrepreneurs possessing richer rural social networks and more substantial financial accumulation for entrepreneurship. By leveraging years of accumulated local connections and traditions of neighborhood mutual aid, elderly entrepreneurs can significantly reduce startup costs, thereby achieving success more readily than their younger counterparts.

Most rural entrepreneurs have no sufficient funds, and scholars find in their studies that the capital element dominated by the financial supply and service system plays a key role in rural entrepreneurship [63]. However, the municipal government of Anhui found that the role of finance is not as big as expected, and the influence of fiscal investment and bank loans is weak, with their roles varying with the region. It may be related to China’s current financial services system, as fiscal investments and bank loans prefer rural infrastructure with universal benefits, such as the construction of roads, bridges, water conservancies, and environmental protection facilities led by village collectives. For rural entrepreneurial activities led by individuals, due to the high level of uncertainty and risk, government and bank policymakers tend to make a verbal or formalized response, with little incentive for proactive and innovative services. Abbasi found out from 240 questionnaires that economic factors have the greatest impact on rural entrepreneurship [64], but the empirical analysis of this study shows that social factors, especially population and innovation, have a stronger impact.

Additionally, Anhui boasts rich regional and rural cultures, which play a crucial yet complex role in the rural entrepreneurship process. On the positive side, culture provides a profound foundation and diverse resources for rural entrepreneurship. For instance, areas like Xidi and Hongcun feature world-renowned Huizhou-style ancient villages, attracting large numbers of domestic and international tourists and thereby spawning numerous rural entrepreneurial activities such as folk customs and specialty souvenir sales. The Jianghuai region of Anhui is rich in folk culture, such as Fengyang flower-drum dance and Chaohu folk songs, enabling farmers to engage in rural entrepreneurship through tourism performances and cultural experience activities. However, it should not be overlooked that some conservative elements contained in the traditional regional culture may constrain rural entrepreneurship. Influenced by traditional agrarian mindsets, some rural areas are slow to accept new ideas and reluctant to take risks on innovative entrepreneurial models, hindering the implementation of projects like smart agriculture initiatives in northern Anhui, where new technologies faced resistance from some villagers. Moreover, the discontinuity in rural cultural heritage poses challenges for rural entrepreneurship, particularly for cultural tourism projects. Many traditional crafts and folk customs lack successors, leaving entrepreneurial projects dependent on these cultural elements struggling with talent shortages. For example, traditional handicrafts like Dingyuan paper-cutting and Huizhou bamboo carving face difficulties in scaling up or industrializing into cultural creative ventures due to a lack of young inheritors, limiting the expansion of rural entrepreneurship in the cultural industry sector.

Overall, the composite effects of different factors indicate that the underlying driving mechanisms of rural entrepreneurship generation and evolution are very complex, and that the hierarchy and interactions of the factors influencing rural entrepreneurship in each region have their own characteristics. Therefore, designers and decision-makers must clarify the nature, intensity, spatial effect and compound effect of the impact factors of rural entrepreneurship in the region, especially the interaction between different factors, before designing rural entrepreneurship management policies. In past research and real-world management efforts, it has been widely hoped by scholars and decision-makers to capture the key influencing factors of rural entrepreneurship. They want the key factors to play a crucial role that may affect the situation as a whole. However, under the combined effects of spatial and composite effects, the hierarchy of factors is subject to large deformations and the nature of the factors’ roles changes in different regions, which makes it difficult to find one or a few key factors that control the process of rural entrepreneurship. The enlightening value of this discovery is that only by mastering the influence mechanism of factors and their transmission and interaction rules can we provide scientific basis for the design of rural entrepreneurial management policy portfolio. That is, the discovery of composite effects can help designers and policymakers figure out the policies to be bundled together, the policies to be avoided from each other, and the new policies that can optimize and adjust the effects of the existing policies (such as positive advantage release or amplification, negative disadvantage suppression or control). It is worth noticing that for rural entrepreneurship in less developed regions, the support of government and actor networks plays a key role [65]. However, the development of rural entrepreneurial activities and its spatial planning and policy design requires the participation of multiple stakeholders. It is not a “one-man show” of the government, but a “chorus” requiring cooperation between the government, entrepreneurs, rural population employees, social organizations, scientific research institutions, industrial chain and upstream and downstream enterprises of the supply chain [66].

## Conclusions

This study innovatively introduced a spatial econometric model to empirically analyze the evolutionary pattern of rural entrepreneurship in Anhui, as well as its influencing factors and its management strategies, reaching the following conclusions:

First, rural entrepreneurship in Anhui shows as increase in spatial heterogeneity, with high, medium and low value areas becoming more concentrated. Therefore, there is a need to delineate geographical zones and design differentiated management policies according to local conditions.

Second, the rural entrepreneurship in Anhui features significant positive spatial autocorrelation, with hot and cold spots showing geographic clustering. Therefore, a rural entrepreneurship alliance is recommended across different regions for resource integration and coordinated development.

Third, rural entrepreneurship in Anhui presents diversified changes, giving rise to rising, falling, stable, fluctuating, inverted U-shaped (first rising and then falling), W-shaped and other patterns. Therefore, classified management should be implemented for rural entrepreneurship according to the actual conditions, and consideration should be given to both quantity management and speed management in policy design.

Fourth, rural entrepreneurship in Anhui is subject to a very complex driving mechanism, with significant spatial and composite effects of factor effects. The factors act as a positive facilitating, negative inhibiting and mixed role in nature, and their action intensity is at key, important and auxiliary levels. The geographical pattern of the factors’ action is characterized by spatial clustering and gradient variation. Factor interactions exhibit synergistic enhancement, with the emergence of super-factor pairs. Therefore, it is necessary to design the policy combination model of rural entrepreneurship management based on the composite and spatial effects of factors.

The innovative and theoretical contributions of this study are mainly in the following three areas. The first is the introduction of spatial econometric modeling for rural entrepreneurship research, which empirically examines the theoretical hypothesis of spatial effects, and provides a new method for academic research on rural entrepreneurship. The second is the discovery that the influence of different factors on rural entrepreneurship has a significant composite effect, and that the superposition of interaction and spatial effect can not only amplify the influence of factors but even change the nature of factors, providing a scientific basis for the design of policy combination system in rural entrepreneurship management practice. The third is the construction of a new research framework that integrates the “evolution model + influencing factor + management strategy” of rural entrepreneurship, achieving mutual adaptation between theoretical research and practical needs, and providing new perspectives for subsequent research.

There are some limitations to this study. For example, restricted by the access to data, the study involved fewer factors of entrepreneurs’ own characteristics to hardly verify the impact of factors such as marriage, family assets, and personal character on rural entrepreneurial activities. Besides, no entrepreneurial quality indicators were available in this study for measuring the rural entrepreneurship index, and it did not include issues such as “entrepreneurship without employment” (many entrepreneurs set up companies without engaging in business operation, making the founded enterprises a tool for obtaining financial subsidies provided by the government for rural entrepreneurship) and entrepreneurial failure in the analysis framework.

## Supporting information

S1 File Rural entrepreneurship and its influencing factors data table. (XLSX)
